# Top-Down Proteomics Identifies Plasma Proteoform Signatures of Liver Cirrhosis Progression

**DOI:** 10.1016/j.mcpro.2024.100876

**Published:** 2024-11-08

**Authors:** Eleonora Forte, Jes M. Sanders, Indira Pla, Vijaya Lakshmi Kanchustambham, Michael A.R. Hollas, Che-Fan Huang, Aniel Sanchez, Katrina N. Peterson, Rafael D. Melani, Alexander Huang, Praneet Polineni, Julianna M. Doll, Zachary Dietch, Neil L. Kelleher, Daniela P. Ladner

**Affiliations:** 1 Proteomics Center of Excellence, Northwestern University, Evanston, Illinois, USA; 2 Northwestern University Transplant Outcomes Research Collaborative (NUTORC), Comprehensive Transplant Center, Feinberg School of Medicine, Northwestern University, Chicago, Illinois, USA; 3 Department of Chemistry, Northwestern University, Evanston, Illinois, USA; 4 Department of Biochemistry and Molecular Genetics, Northwestern University Feinberg School of Medicine, Chicago, Illinois, USA

**Keywords:** liver cirrhosis, top-down proteomics, proteoforms, LC-MS/MS, fibrinogen alpha chain, haptoglobin, apo A-I, mass spectrometry

## Abstract

Cirrhosis, advanced liver disease, affects 2 to 5 million Americans. While most patients have compensated cirrhosis and may be fairly asymptomatic, many decompensate and experience life-threatening complications such as gastrointestinal bleeding, confusion (hepatic encephalopathy), and ascites, reducing life expectancy from 12 to less than 2 years. Among patients with compensated cirrhosis, identifying patients at high risk of decompensation is critical to optimize care and reduce morbidity and mortality. Therefore, it is important to preferentially direct them towards specialty care which cannot be provided to all patients with cirrhosis. We used discovery top-down proteomics to identify differentially expressed proteoforms (DEPs) in the plasma of patients with progressive stages of liver cirrhosis with the ultimate goal to identify candidate biomarkers of disease progression. In this pilot study, we identified 209 DEPs across three stages of cirrhosis (compensated, compensated with portal hypertension, and decompensated), of which 115 derived from proteins enriched in the liver at a transcriptional level and discriminated the three stages of cirrhosis. Enrichment analyses demonstrated DEPs are involved in several metabolic and immunological processes known to be impacted by cirrhosis progression. We have preliminarily defined the plasma proteoform signatures of cirrhosis patients, setting the stage for ongoing discovery and validation of biomarkers for early diagnosis, risk stratification, and disease monitoring.

## Highlights

• Performed a Top-down LC-MS/MS analysis on the plasma of patients with three stages of liver cirrhosis.
• Identified >2800 plasma proteoforms (PFR) in different stages of the disease.
• Identified 209 differentially expressed proteoforms (DEP) in different stages of the disease.
• Identified DEP profiles able to potentially distinguish early from late stages of the disease.
• Identified DEPs from three proteins as candidate biomarkers of liver cirrhosis progression.

## Graphical Abstract

**Figure:**
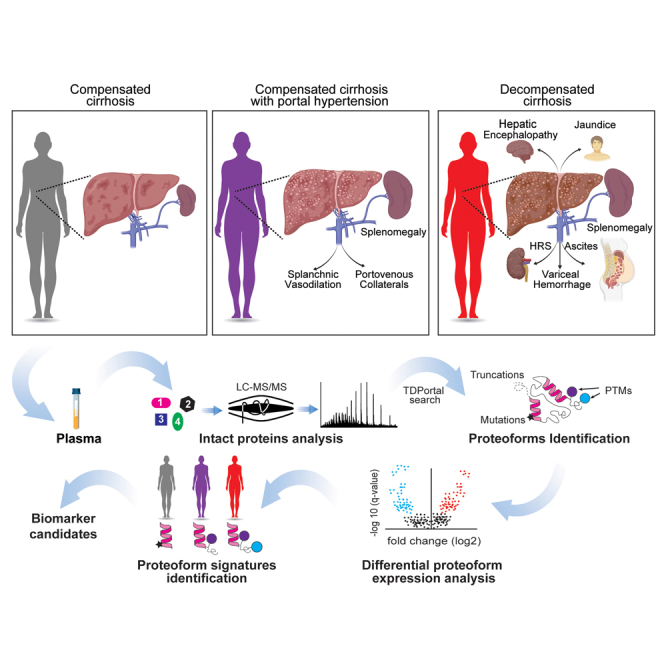

## In Brief

Cirrhosis affects 2 to 5 million Americans, with many developing severe complications. Identifying patients at high risk is crucial. Using top-down proteomics, we found 209 differentially expressed proteoforms (including 115 proteoforms of liver proteins) in plasma samples from patients across three cirrhosis stages (compensated, compensated with portal hypertension, and decompensated). These findings highlight potential biomarkers for disease progression, aiding early diagnosis and risk stratification. This study lays the groundwork for future biomarker discovery and validation to improve patient care.

The prevalence of cirrhosis and end-stage liver disease is increasing and poses a significant public health burden, in terms of hospitalizations, cost, and mortality (1, 2, 3, 4, 5). In fact, mortality of cirrhosis is comparable to diabetes and pneumonia and surpasses the mortality associated with most cancers (6, 7). Cirrhosis is the result of liver injury most frequently caused by alcohol use disorder, metabolic dysfunction-associated liver disease [previously, non-alcoholic fatty liver disease], and hepatitis C virus, along with other less frequent etiologies (*e.g.*, biliary, hepatitis B). Most patients with cirrhosis have compensated chronic disease, which carries a life expectancy of ∼12 years (8). However, as cirrhosis progresses, intrahepatic structural changes lead to diversion of blood flow to the heart through alternate small blood vessels, rather than the liver. This process of porto-venous collateral formation and splanchnic vasodilation is called portal hypertension (pHTN) (9, 10, 11). As liver disease progresses, pHTN can turn into decompensated cirrhosis, considered as the occurrence of confusion (hepatic encephalopathy), ascites, and gastrointestinal bleeding. Decompensated cirrhosis is associated with significant morbidity, high rates of hospitalizations, and decreased life expectancy of 1.8 years. On average, one in 10 patients with cirrhosis experience a decompensating event every year (8). With the increasing prevalence of metabolic syndrome and alcohol use disorder, the two major causes of cirrhosis, cirrhosis-related morbidity, and mortality are expected to rise (5, 12).

Early diagnosis, optimal disease management, and identification of patients who are at high risk of decompensation is critical to provide patients with targeted and timely interventions, mitigate disease progression, and decrease morbidity and mortality (13, 14). While only experimental drugs exist to mitigate disease progression (15, 16), timely specialty care can optimize the disease course. However, with 2-5M adults affected by cirrhosis and limited resources including specialists, identifying those at highest risk for disease progression is paramount for optimal triage and outcomes. Presently, such diagnostic tools are not widely available. Hepatic vein pressure gradients have been shown to be a marker of decompensation (17), but the procedure is invasive and may not be available at all centers. Clinical risk scores may hold some prognostic value, but the majority were initially established to predict mortality and not disease progression (18, 19). To this end, noninvasive and reliable diagnostic tools to identify and predict identify those at highest risk are required. Top-down proteomics (TDP) can be used to develop predictive markers of disease progression by leveraging modified proteins that drive mechanisms of disease. TDP can characterize proteins present in biological systems by determining the molecular composition of all forms of a given protein, so called “proteoforms” (20, 21, 22, 23, 24). Proteoforms (PFRs) arise due to genetic variation in protein coding regions, alternative splicing, and/or posttranslational modifications (PTMs) (25). It is estimated that the human proteome contains >50 million unique PFRs compared to ∼20,300 gene-encoded proteins (26). As a result, by delivering information at the proteoform level, TDP captures the complexity of a biological system better than that of traditional peptide-based proteomics approaches, which only provide data at the protein level. We have recently demonstrated the importance of PFRs as biomarkers in the context of liver transplantation with the identification of PFR signatures of hematopoietic stem cells able to distinguish acute rejection from normal graft function (27). PFRs specific for cirrhosis, especially those specific to different stages of cirrhosis, have not been described, and their identification could greatly improve the diagnosis and treatment of those at highest risk for progression.

In this study, we applied a TDP approach to identify PFRs present in the plasma of patients with three different stages of cirrhosis [compensated (stage I)/compensated + pHTN (stage II)/decompensated (stage III)]. We found that patients with different stages of cirrhosis have unique blood proteoform profiles that impact important pathophysiological pathways and cellular processes. Our findings create a framework for applying proteoform-based analysis to biomarker discovery in the cirrhosis population and can provide mechanistic insight to the disrupted processes as the disease progresses.

## Experimental Procedures

### Human Subject Selection and Plasma Collection

Peripheral blood was collected from 30 subjects with cirrhosis as part of clinical cirrhosis care at Northwestern Medicine who had compensated cirrhosis (stage I, n = 9), compensated cirrhosis + pHTN (stage II, n = 10), and decompensated cirrhosis (stage III, n = 11). Plasma was then isolated and stored at −80 °C until protein extraction. Samples from adults (>18 years) with 1) compensated cirrhosis, 2) compensated cirrhosis + pHTN, and 3) decompensated cirrhosis were used. Diagnosis of cirrhosis and stage of cirrhosis was determined by careful medical record review (JMS, PP, DPL), including Fibroscan, magnetic resonance elastography, or biopsy. Decompensation was defined as the presence of hepatic encephalopathy, ascites, hepatorenal syndrome, spontaneous bacterial peritonitis, and history of variceal gastrointestinal bleeding (28, 29). pHTN was diagnosed either by imaging, presence of thrombocytopenia (platelets <100), or evidence of esophageal/gastric varices or portal hypertensive gastropathy on surveillance/diagnostic endoscopy (29, 30). Demographic, clinical characteristics, and laboratory values (complete blood count (CBC), liver enzymes, basic chemistries, and International Normalized Ratio) were collected for each subject at the time of lab draw (Fig. S1). The Model for End Stage Liver Disease Sodium (MELD-Na) was calculated for each subject.

### Study Approval

This study was conducted in compliance with the NIH guidelines for studies involving human subjects and abides by the principles of the Declaration of Helsinki. The Northwestern Institutional Review Board (IRB) issued a Waiver of Consent and approved this study under STU00216399.

### Plasma Protein Extraction and Fractionation

Protein concentration in the plasma was determined by a bicinchoninic acid protein assay kit (Thermo Fisher Scientific) following the manufacturer instructions. Briefly, 500 μg of plasma proteins were resuspended in 4X NuPAGE loading buffer (Thermo Fisher Scientific) with 50 mM DTT and boiled at 95 °C for 10 min. We applied polyacrylamide gel–based prefractionation for the analysis of intact proteoforms and protein complexes by mass spectrometry (PEPPI-MS) to analyze plasma samples (31, 32). PEPPI-MS utilizes passive extraction of gel fractionated proteins with a molecular weight <30 kDa prior to MS analysis (31). We utilized the workflow of PEPPI-MS analysis on cirrhotic patient plasma samples using a simple SDS page gel electrophoresis for proteoform fractionation and recovery. Size-based fractionation systems such as gel-eluted liquid fraction entrapment electrophoresis now require custom workflows for preparative electrophoresis, as commercial gel-eluted liquid fraction entrapment electrophoresis systems have been discontinued (33, 34). Prefractionation methods of high-resolution separation of complex mixtures using serial-size exclusion chromatography was not used here (35). Proteins were fractionated on a NuPAGE 4 to 12% Bis-Tris gel (Thermo Fisher Scientific) for 10 min at 70 V and 15 min at 150 V. The gel was briefly rinsed with deionized water and gel sections with proteins with a molecular weight below 30 kDa were excised from the gel and transferred to a Protein LoBind tube (Eppendorf) containing 100 mM ammonium bicarbonate, pH 9 with 0.1% SDS. Proteins were extracted from the gel by crushing it with a pestle and shaking the slurry for 20 min at 1400 rpm. Gel debris were removed with 0.45 μm centrifugal filters. The protein fractions were methanol/chloroform/water precipitated by using a slightly modified Wessel and Flügge’s method (36) of LC-MS buffer A (5% acetonitrile, 94.8% water, and 0.2% formic acid) and subjected to liquid chromatography with tandem mass spectrometry (LC-MS/MS).

### LC-MS/MS Analysis

Proteoforms separation was conducted on a Dionex Ultimate 3000 (Thermo Fisher Scientific) by using a trap/column system consisting of the following: 1) a trap (150 μm I.D. × 2.5 cm length) packed in-house with ReproSil C4, 3 μm particles (Dr Maisch), 2) a FlowChip column (25 cm in length × 100 μm i.d. obtained from New Objective). For the separation, the flow rate was set at 1 μl/min and the following gradient was used: 5% B (5% water, 94.8% acetonitrile, and 0.2% formic acid) from 0 to 10 min, 20% B at 15 min, 55% B at 100 min, 95% B from 103 to 110 min, 5% B at 115 to 120 min.

The LC is in line with the Orbitrap Eclipse (Thermo Fisher Scientific) mass spectrometer operating in “protein mode” with 2 mTorr of N_2_ pressure in the ion routing multipole. Transfer capillary temperature was set at 320 °C, ion funnel RF was set at 50%, and a 15 V of source CID was applied. MS (1) spectra were acquired at 120,000 of resolving power (at *m/z* 200), AGC target value of 1000%, 100 ms of maximum injection time, and 1 μscan. Data-dependent top-N-2 s MS (2) method used 32 NCE for HCD to generate fragmentation spectra acquired at 60,000 resolving power (at *m/z* 200), with target AGC values of 2000%, 600 ms maximum injection time, and 1 μscan. Precursors were quadrupole isolated using a 3 *m/z* isolation window, dynamic exclusion of 60 s duration, and threshold of 1 × 10^4^ intensity. Each sample was acquired in triplicate for a total of 90 runs.

## Experimental Design and Statistical Rationale

A total of 30 biological replicates (state I: n = 9, state II: n = 10, state III: n = 11) were analyzed in triplicate (*i.e.*, three technical replicates each) using LC-MS/MS for TDP.

### Data Analysis for LC-MS/MS

The raw data files were processed with the publicly available workflow on TDPortal (https://portal.nrtdp.northwestern.edu, Code Set 4.0.0) that performs mass inference, searches a database of human proteoforms derived from Swiss-Prot (June 2020) with curated histones, and estimates a conservative, context-dependent 1% false discovery rate at the protein, isoform, and proteoform levels (37).

### Statistics and Functional Enrichment Analysis

Data derived from randomized biological & technical replicates were used to assess variability associated with sample processing. From the aggregated data sets, a batch effect was observed and corrected with batch standardization (z-score) and statistical analysis was conducted using SAS (Cary, NC) with custom scripts. The appropriate hierarchical linear statistical model was applied for quantitation using SAS PROC MIXED (SAS Institute). Normalized and batch corrected ion intensities from the data files were run through a nested ANOVA process to measure both effect size and significance in differential proteoform expression between the three patients’ groups. Specific proteoform signatures were then correlated with decompensated outcomes. *p*-values obtained from the mixed model linear regression were adjusted (Benjamini & Hochberg algorithm) to control the false discovery rate induced by multiple comparison tests. PFRs with adjusted *p* value < 0.05 and fold changes higher than 1.5 or less than 0.66 were considered differential expressed proteoforms (DEPs). A Partitioning Around Medoids (38) unsupervised algorithm (‘cluster::Pam’Rfunction) was applied to group PFRs based on their expression changes between different disease stages. The number of clusters (k) was manually set to three, as it was the number of clusters that best showed changes in disease progression. To visualize clusters based on differential expressions and changes in specific PFR signatures, we created heatmaps using the ‘ComplexHeatmap’ R package. For creating the heatmap including clinical biomarkers, we used standardized clinical biomarkers across samples using z-score normalization. To know whether the PFR modifications were significantly different between groups of DEPfr, we applied Fisher exact tests followed by an adjustment of *p*-values (Benjamini & Hochberg). Proteins having at least one deregulated PFR were selected to perform Gene Ontology enrichment analysis based on Biological Process (BP), and the analysis was carried out using ‘clusterProfiles’ R package.

## Results

### Discovery TDP Analysis of Plasma Characterizes Blood Proteoform Profiles in Cirrhosis Patients

**Fig. 1:**
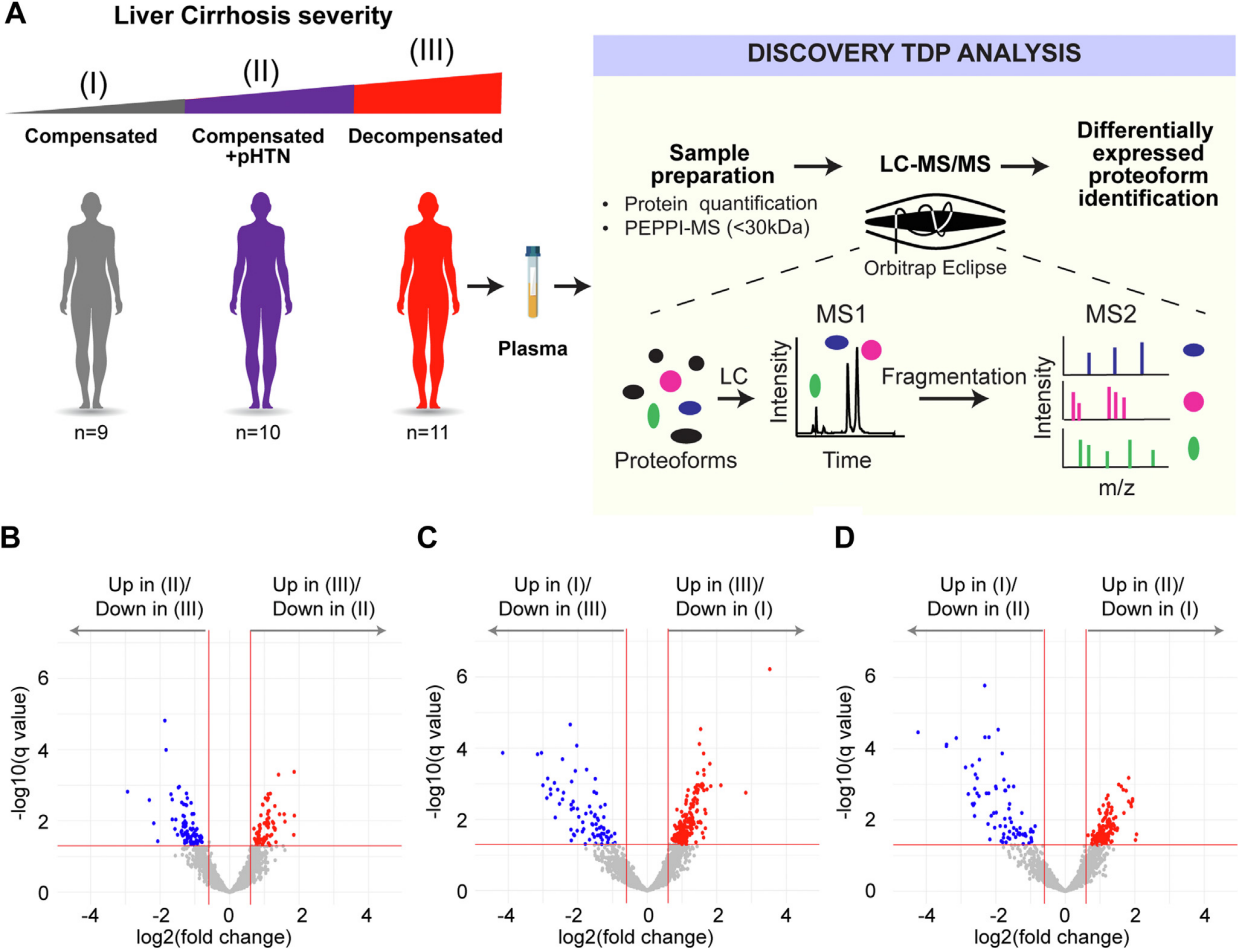
**Workflow used to identify differentially expressed proteoforms in patients with cirrhosis.***A*, plasma was extracted from cirrhosis patients with three stages of disease—compensated (I), compensated with portal hypertension (II), and decompensated cirrhosis (III). Plasma fractions of 30 kDa were then subjected to top-down proteomics (TDP) to identify DEPs across disease stages. *B*–*D*, volcano plots showing differentially expressed proteoforms (DEPs) in the plasma from patients with (*B*) decompensated cirrhosis (III) *versus* compensated cirrhosis with portal hypertension (II), (*C*) decompensated cirrhosis (III) *versus* compensated cirrhosis (I), and (*D*) compensated cirrhosis with portal hypertension (II) *versus* compensated cirrhosis (I). A false discovery rate (FDR) threshold of 5% for differential expression was used.

The cohort included 30 subjects—9 (30%) with compensated cirrhosis (stage I), 10 (33.3%) with compensated cirrhosis + pHTN (stage II), and 11 (36.7%) with decompensated cirrhosis (stage III) (Fig. 1*A*). Demographics and clinical data are shown in Table 1. The mean age of patients was 59.7 years (stage I: 63.7, stage II: 55.5, stage III: 61.9). 40% (n = 12) of patients were female (stage I: 55.6%, stage II 40.0%, stage III: 27.3%) and 56.7% (n = 17) were White. The main etiology of cirrhosis was alcohol use disorder in 60% (n = 18). The mean MELD-Na was 11.5 (stage I: 9.9, stage II: 10.0, and stage III: 14.1). Detailed laboratory findings are shown in Supplemental Fig. S1.

**Table 1:** Characteristics of the patient cohort

Patient characteristic	Total (n = 30)	Compensated (n = 9)	Compensated w/pHTN (n = 10)	Decompensated (n = 11)	*p*-value
Age, mean (SD)	59.73 (12.34)	63.67 (11.18)	55.51 (10.54)	61.89 (14.18)	0.314
Female, n (%)	12 (40)	5 (55.6)	4 (40.0)	3 (27.3)	0.612
BMI, mean (SD)	29.14 (5.55)	31.14 (6.49)	28.63 (3.21)	27.99 (6.42)	0.336
MELD-Na, mean (median [IQR])	11.47 (4.62)	9.89 (3.18)	10.00 (3.94)	14.09 (5.30)	0.162
Race/Ethnicity, n (%)					0.206
Asian	0 (0.0)	0 (0.0)	0 (0.0)	0 (0.0)	
Black or African American	3 (10)	2 (22.2)	1 (10.0)	0 (0.0)	
Hispanic or Latino	10 (33.33)	4 (44.4)	3 (30.0)	3 (27.3)	
Other	2 (6.67)	0 (0.0)	0 (0.0)	2 (18.2)	
White	17 (56.67)	3 (33.3)	6 (60.0)	8 (72.7)	
Etiology, n (%)					<0.01
AIH	3 (10)	0 (0.0)	2 (20.0)	1 (9.1)	
PBC	1 (3.33)	0 (0.0)	0 (0.0)	1 (9.1)	
ETOH	18 (60.0)	5 (55.6)	5 (50.0)	8 (72.7)	
HBV	1 (3.33)	1 (11.1)	0 (0.0)	0 (0.0)	
HCV	8 (26.67)	4 (44.4)	3 (30.0)	1 (9.1)	
MASH	4 (13.33)	1 (11.1)	1 (10.0)	2 (18.2)	

pHTN, portal hypertension; AIH, autoimmune hepatitis; PBC, primary biliary cholangitis; ETOH, alcohol-associated liver disease; HBV, hepatitis B virus; HCV, hepatitis C virus; MASH, metabolic dysfunction associated steatohepatitis; BMI, body mass index; MELD-Na, Model for End-Stage Liver Disease with Sodium.

We first conducted an untargeted quantitative TDP analysis of 0 to 30 kDa fractions of plasma (Fig. 1*A*). Within the entire cohort, 2867 proteoforms from 99 proteins were captured (Supplemental Table S1), of which, 47% were identified at level 1, with PTMs being clearly assigned to specific sites with low ambiguity (39). Modifications of proteoforms mainly included C- and N- terminus truncations (Supplemental Fig. S2). Phosphorylation was the most frequent PTM (Supplemental Fig. S2). Other common PTMs included monoacetylation, alpha-amino acetylation, S-nitrosyl-L-cysteine, n-pyruvic acid 2-iminyl-L-valine, monomethylation, monohydroxylation, L-gamma-carboxyglutamic acid, and 2-pyrrolidone-5-carboxylic acid (Supplemental Fig. S2). Monoisotopic masses of the proteoforms ranged from 1.6 to 38.9 kDa, with a median value of 15.3 kDa (Supplemental Fig. S3).

### Proteoforms are Differentially Expressed in Stages of Cirrhosis and are Involved in Biologically and Clinically Relevant Processes

We then separated samples according to their diagnostic stage (I-III) (Fig. 1, *B*–*D*) and identified a total of 663 DEPs from 48 proteins. Like the ∼3000 total proteoforms, modifications of DEPs mainly included truncations, phosphorylation, and acetylation (Supplemental Fig. S2). Pairwise comparisons between the three stages of cirrhosis showed the following: 1) 74 upregulated and 83 downregulated DEPs comparing stage III to stage II (Fig. 1*B*), 216 upregulated and 79 downregulated DEPs comparing stage III to stage I (Fig. 1*C*), and 136 upregulated and 75 downregulated DEPs comparing stage II to stage I (Fig. 1*D*). In our differential expression analysis, we identified several proteoforms from hemoglobin subunits. As hemoglobin presence could be due to sample handling and preparation, hemoglobin proteoforms were omitted from further analyses. Therefore, downstream analyses were done with 209 proteoforms from 44 proteins (Supplemental Table S2). Of the 209 DEPs, 82% were identified at level 1, with unambiguous PTM assignments (39) (Supplemental Table S2). In the pairwise comparisons shown in Supplemental Figs. S4–S6, we found that some PFRs from a single protein were upregulated while other PFRs from the same protein were downregulated. For example, we identified a total of 14 DEPs for fibrinogen alpha chain, of which five were downregulated and nine were upregulated in stage III *versus* stage II (Supplemental Fig. S4), 11 DEPs for apolipoprotein A-I, of which two were downregulated and nine were upregulated in stage III *versus* stage I (Supplemental Fig. S5), and two DEPs for ceruloplasmin, of which one was upregulated and one was downregulated in stage II *versus* stage I (Supplemental Fig. S6). We next analyzed the DEP modifications found in the three pairwise comparisons (Supplemental Fig. S7*A*). DEP modifications in the comparison between stages III and I included mostly truncations (83%), followed by monohydroxylation (7%), alpha-amino acetylation (6%), and phosphorylation (2%). Both L-gamma-carboxyglutamic acid and 2-pyrrolidone-5 carboxylic acid residues occurred in 1% of the modifications. In the comparison between stages II and I, the top three modifications were again truncation (75%), monohydroxylation (11%), and alpha-amino acetylation (9%). Phosphorylated, monomethylated, L-gamma-carboxyglutamic acid, and 2-pyrrolidone-5 carboxylic acid residues each occurred in 1% of the modifications. Finally, in the comparison between stages III *versus* II, we found 63% of PTMs were truncations followed by phosphorylation (11%), alpha-amino acetylation (11%), monohydroxylation (9%), carboxylation of glutamic acid residues (4%), monoacetylation (1%), and pyrrolidone-5 carboxylic acid residues (1%). We then compared the number of each modification in the three sets of DEPs (sets: stage III *versus* I, II *versus* I, III *versus* II) and found that phosphorylation was the only PTM to be statistically significantly different between the three sets (Supplemental Fig. S7*B*).

**Fig. 2:**
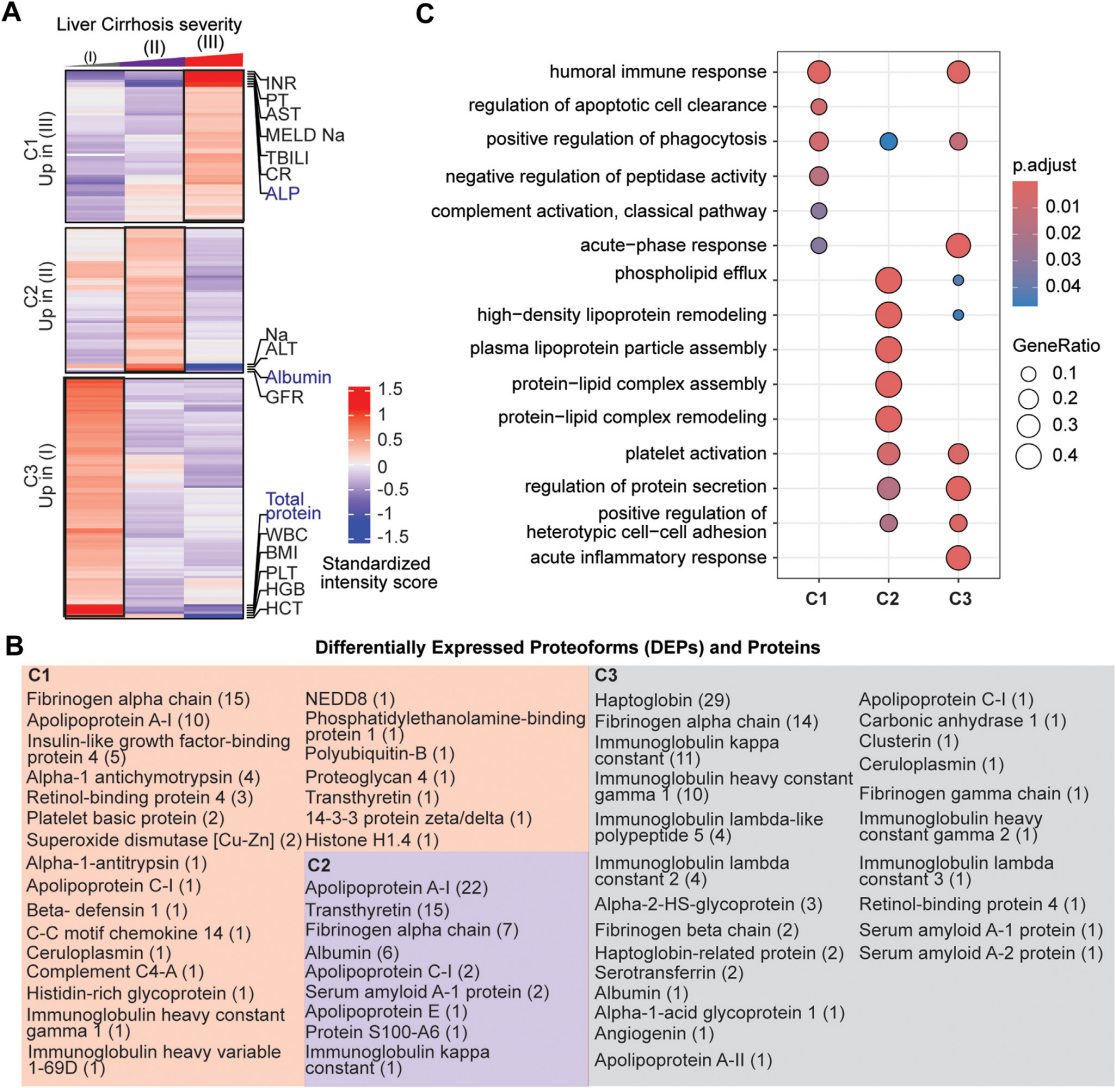
**Proteoforms found to be differentially expressed across stages of cirrhosis.***A*, cluster distribution of differentially expressed proteoforms (DEPs) and currently used clinical markers of disease severity. Clinical markers highlighted in *blue* are significantly different between stages. Cluster 1 (C1) included DEPs upregulated in stage III disease, C2 included DEPs upregulated in stage II disease, and C3 included DEPs upregulated in stage I disease. *B*, proteins from which DEPs derived are shown within each cluster. The number of proteoforms of a given protein is shown in parentheses beside the protein name. For example, in C1, the fibrinogen alpha chain protein had 15 DEPs identified. *C*, biological process enrichment analyses on proteins identified in each cluster. The level of significance is represented by the color of the bar with scale, while the gene ratio is indicated by the diameter of the *circle*.

DEPs were then clustered based on their regulation patterns in each stage (Fig. 2, *A* and *B*). Laboratory test values from clinical evaluation were also included in the clustering to find possible associations between DEPs and clinical markers of disease severity. We also performed gene ontology based on BP enrichment analyses to correlate PFR profiles with affected BP. Cluster 1 (C1) included DEPs that were significantly upregulated in decompensated disease (stage III). This cluster includes 57 DEPs from 23 proteins. The proteins with the highest number of DEPs in C1 were fibrinogen alpha chain with 15 DEPs, apolipoprotein A-I with 10 DEPs, and insulin-like growth factor-binding protein 4 with 5 DEPs. Pathway enrichment analysis indicated that C1 DEPs derived from proteins involved in several BP, including the humoral immune response, apoptosis regulation, complement activation, phagocytosis, and negative regulation of peptidase activity (Fig. 2*C* and Supplemental Table S3). In addition, C1 DEPs clustered with several clinical markers, including alkaline phosphatase, which was significantly higher in stage III (Supplemental Fig. S1), total bilirubin, which was significantly increased between stage II and III, and MELD-Na, which was significantly and appropriately higher in stage III than stage I (MELD-Na is a marker of disease severity). Other clinical markers included in this cluster were International Normalized Ratio, prothrombin time, aspartate aminotransferase, and creatinine, although their increase in stage III was not statistically significant.

Cluster 2 (C2) included 57 DEPs upregulated in stage II. The PFRs derived from nine proteins involved in phospholipid efflux and processes regulated by apolipoproteins (Fig. 2*C* and Supplemental Table S3). The proteins with the highest number of DEPs were apolipoprotein A-I, transthyretin, and fibrinogen alpha chain with 22, 15, and 7 DEPs, respectively. Clinical markers in this cluster included glomerular filtration rate, Sodium (Na), alanine aminotransferase, and albumin. Interestingly, six out of seven albumin PFRs detected in the TDP discovery analysis were found in this cluster together with albumin protein, which was analyzed by routine clinical laboratory testing (Supplemental Fig. S1). Both albumin PFR and protein levels were significantly higher in the plasma of patients with stage II and lower in those with stage III. In addition, fold changes obtained for the albumin protein (laboratory results) and albumin PFRs were comparable despite using two distinct approaches, confirming the validity of the TDP discovery analysis.

Cluster 3 (C3) included 95 DEPs upregulated in stage I. These DEPs derived from 24 proteins associated with the immune system BP, such as the acute inflammatory response, acute-phase response, and platelet activation. Other processes enriched in C3 were regulation of protein secretions and cell-cell adhesion (Fig. 2*C* and Supplemental Table S3). In this cluster, haptoglobin was the protein with the highest number of DEPs (29) followed by fibrinogen alpha chain (16). Interestingly, clinical markers related to the immune system, such as white blood cell count, platelet count, and total proteins were grouped in C3. However, only the total protein value changed significantly between stages I *versus* II and I *versus* III (Supplemental Fig. S1).

Overall, clusters containing DEPs upregulated in the late stages of the disease (C1 and C2) were mainly from fibrinogen alpha chain, transthyretin, and apolipoprotein A-I proteins (Fig. 2*B*). However, another important group of DEPs from fibrinogen alpha chain (14 PFRs) were upregulated in stage I and clustered with haptoglobin in C3. In terms of BP, DEPs upregulated in the early stage of the disease were involved in cellular defense mechanisms, while those upregulated in the late stages of cirrhosis were associated with cell death. Percentages of DEP modifications differed in the three clusters as shown in Supplemental Fig. S8*A*. DEPs in C3 had the highest percentage of truncations (90% *versus* 77% and 54% of DEPs observed in C1 and C2, respectively). Phosphorylation was higher in C2 (10%) than C1 (3%) and not observed in DEPs in C3. In addition, C2 had a higher percentage of alpha-amino acetylated (C1 = 6%, C2 = 13%, C3 = 6%), monohydroxylated (C1 = 11%, C2 = 17%, C3 = 1%), and L-gamma carboxyglutamic acid (C1 = 0%, C2 = 6%, C3 = 0%) residues. C2 did not contain any 2-pyrrolidone-5-carboxylic acid modifications, which was instead observed in 1% of C1 and C3 DEPs. Monohydroxylation was only observed in C1 and monomethylation in C3. Finally, the comparison of the number of each modification in the three clusters indicated a significant variation in the types of PFR modifications at the level of phosphorylated, monohydroxylated, alpha-amino acetylated, and L-gamma-carboxyglutamic residues (Supplemental Fig. S8*B*).

### Liver-Derived Proteoforms may be Candidate Biomarkers for Cirrhosis Progression

**Fig. 3:**
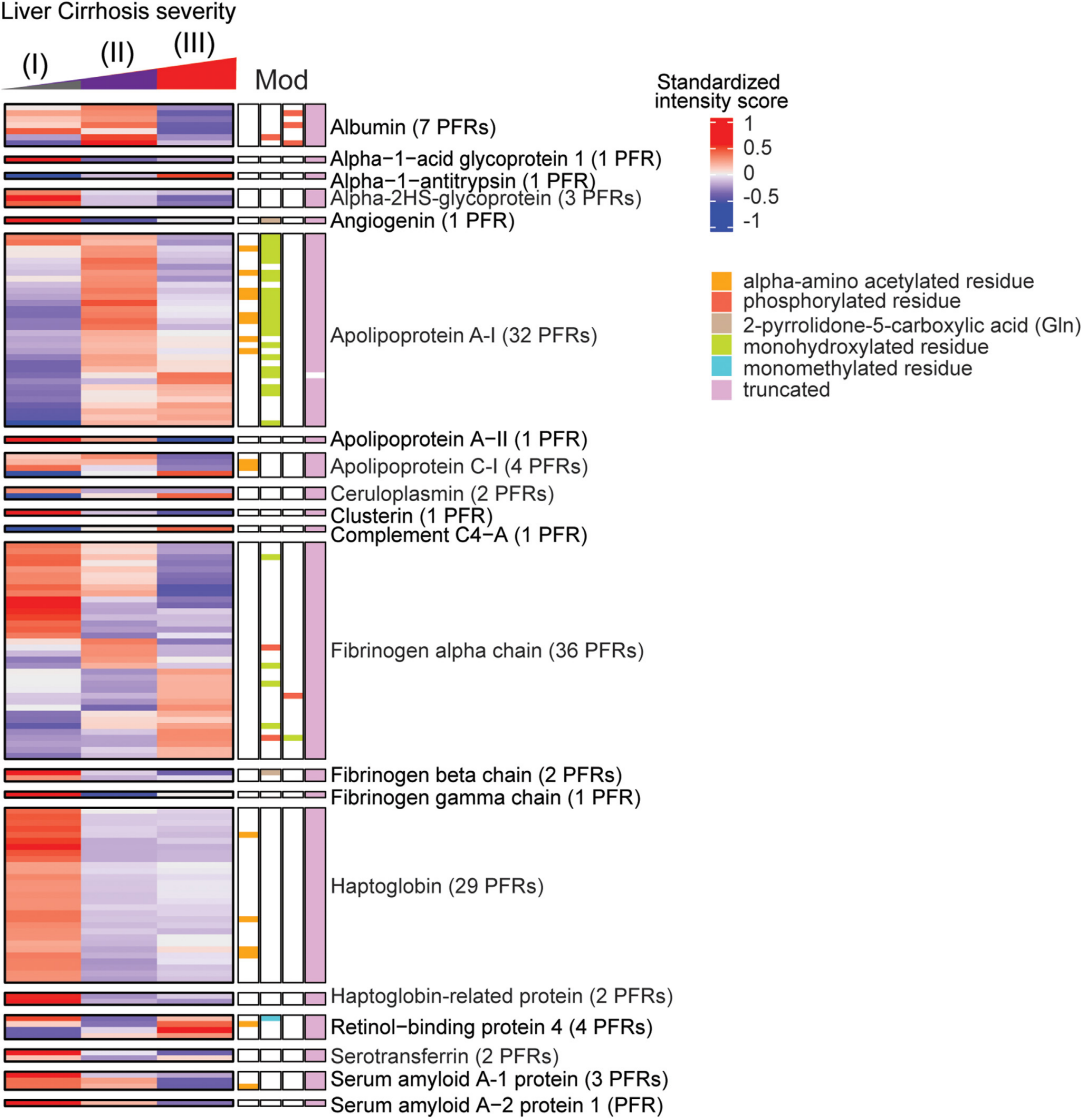
**Heatmaps of quantified proteoforms of proteins enriched in the liver at transcriptional level.** Associated proteoform modifications are shown and defined by the figure legend. The proteins and number of identified proteoforms derived from each protein (in parenthesis) are indicated on the *right side* of the heatmap. MOD, modification; PFR, proteoform.

Heatmaps were generated for the DEPs identified above but given that cirrhosis is associated with ongoing damage to hepatocytes, we primarily focused on PFRs derived from proteins that are enriched in the liver tissue (https://www.proteinatlas.org) at a transcriptional level as they may be the most sensitive to differences between stages (Supplemental Figs. S4–S6). Heatmaps of these liver-enriched DEPs were created for each stage of cirrhosis, along with their modifications, including truncations and a variety of PTMs (Fig. 3). As the majority of DEPs discriminating the different stages of the disease derived from fibrinogen alpha chain, apolipoprotein A-1, and haptoglobin, we analyzed those PFRs in more detail (Fig. 3 and Supplemental Fig. S9). Fibrinogen alpha chain PFRs were found in all clusters. Specifically, 14 PFRs were detected in C1, 7 in C2, and 15 in C3. The canonical sequence of fibrinogen alpha chain includes 866 amino acids. All the identified PFRs were truncated forms of the protein missing both the N- and C-termini. In C1 (PFRs upregulated in stage III), PFR sequences were generally longer with an average of 126 amino acids. The average length in the other clusters was 58.86 and 58 for C2 and C3, respectively. This suggests the presence of shorter truncations in early stages of the disease. In terms of PTMs, the majority was found in C1. In this cluster, we detected three PFRs with monohydroxylation and three phosphorylated PFRs. In C2, PTMs included one monohydroxylation and one phosphorylation. C3 (PFRs upregulated in stage I) contained one PFR with monohydroxylation. Of the 32 apolipoprotein A-I PFRs, 22 were in C2 and 10 in C3. The majority were truncated and had 1) no additional PTM, 2) alpha-amino acetylation, or 3) methionine oxidation. A proline 27 to histidine point mutation was also found in two PFRs in C2. Haptoglobin PFRs included both truncations of the canonical protein (P00738-1) and of an isoform that lacks amino acids 38 to 96 (P00738-2). In addition, we found mutations in 10 truncated PFRs of haptoglobin (P00738-2), of which eight had an aspartic acid residue replacing asparagine in position 70 and two with lysine at the place of glutamic acid in position 71. In terms of PTMs, we found that four of the haptoglobin PFRs were acetylated at the N-terminus. Although not described in detail, we also identified DEPs deriving from proteins that are not enriched in the liver tissue at a transcriptional level (Supplemental Figs. S4–S6). Heatmaps of nonliver-enriched PFRs are displayed in Supplemental Fig. S10.

**Fig. 4:**
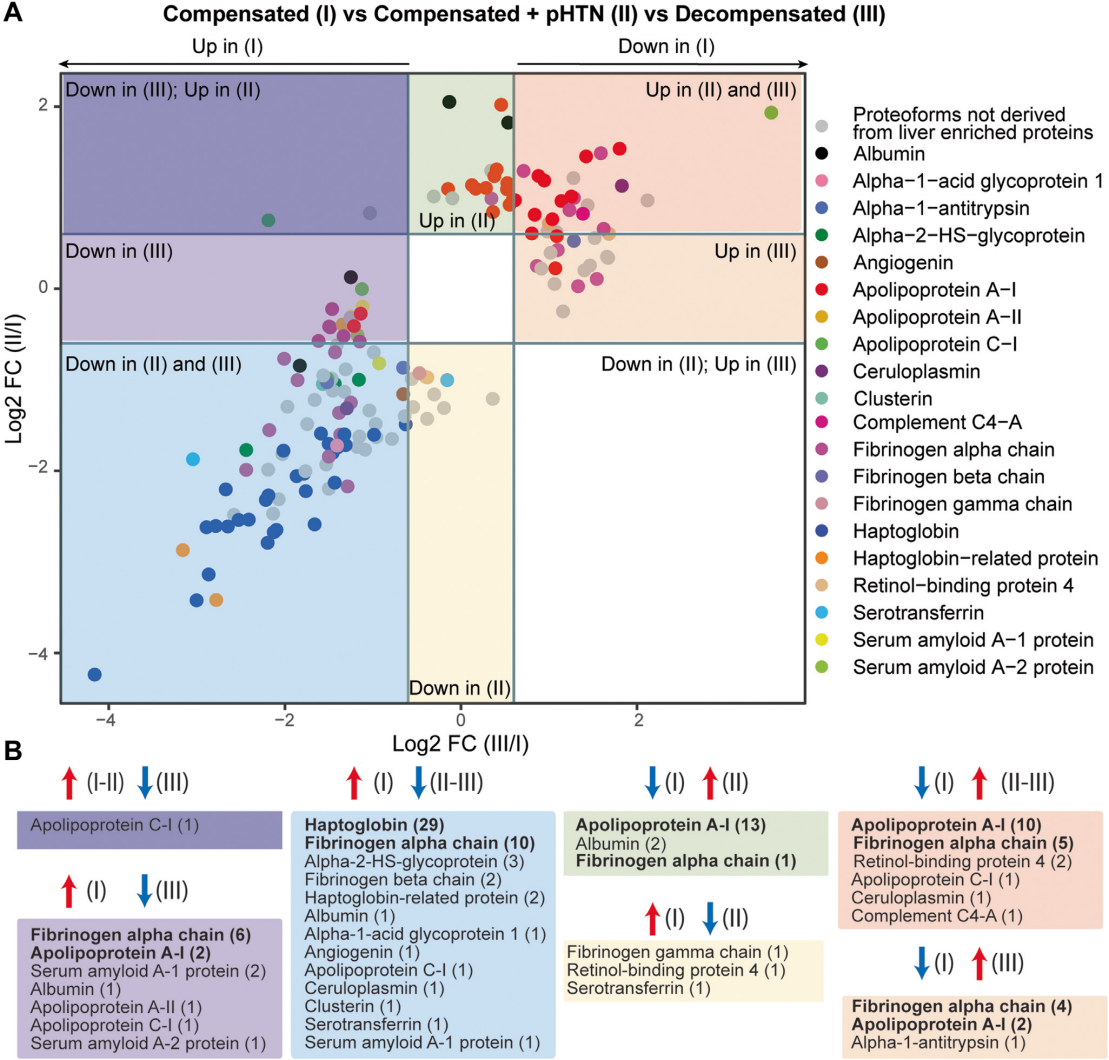
**Display of proteoforms stratified by expression in each stage of cirrhosis.***A*, differentially expressed proteoforms (DEPs) in stages II and III disease with stage I disease (compensated) used as the reference. Proteoforms upregulated in stage II and III (*red square*), upregulated only in stage III (*orange square*), downregulated in stages II and III (*blue square*), downregulated in stage III only (*light purple square*), downregulated in stage III but upregulated in stage II (*dark purple square*), upregulated only in stage II (*green square*), and downregulated only in stage II (*yellow square*). Proteoforms deriving from proteins enriched in liver are highlighted and grouped by color according to the figure legend. *B*, profiles of proteoforms upregulated in stages I and II and downregulated in stage III (*dark purple*), upregulated in I and downregulated in III (*light purple*), upregulated in I and downregulated in II and II (*light blue*), downregulated in I and upregulated in II (*green*), upregulated in I and downregulated in II (*yellow*), downregulated in I and upregulated in II and III (*red*), and downregulated in I and upregulated in III (*orange*). The proteins from which DEPs derive and the relative number of proteoforms (in parenthesis) are shown. Proteins with the highest number of DEPs for each group are shown in bold.

To more clearly define PFR signatures that correlated with disease progression, we created profiles of DEPs that could discriminate stage I (compensated) from stages II and III of cirrhosis. Comparisons of PFR expression were performed across cirrhosis stages, with compensated disease (stage I) used as the reference group (Fig. 4*A*). This analysis identified 167 DEPs (from 37 proteins), of which 115 DEPs derived from 20 proteins enriched in the liver at a transcriptional level (Fig. 4*B*). Tables containing detailed information about these 115 DEPs and their modifications are included in Supplemental Figs. S11–S13 and Supplemental Table S2. Apolipoprotein C-I- PFR#16559 was the only DEP found downregulated in stage III but upregulated in stages I and II (Fig. 4, *A* and *B*, and S11, Dark purple square). There were 14 DEPs downregulated in stage III of the disease compared to stage I. These included six DEPs from fibrinogen alpha chain, 2 from apolipoprotein A-I and serum amyloid A-1 protein, and one from albumin, apolipoprotein A-II, apolipoprotein C-I, and serum amyloid A-2 protein (Fig. 4, *A* and *B*, Light purple square). Both apolipoprotein A-I PFRs contained oxidized methionine residues at positions 110 and 136 and had N-terminus truncations at two different positions (residues 67 and 35, respectively) (Supplemental Fig. S11). None of the fibrinogen alpha chain PFRs had a PTM, but all contained both C- and N-termini truncations. The average length of truncation was 55.83 amino acids. Three DEPs were specifically downregulated in stage II of the disease and derived from serotransferrin, retinol-binding protein 4, and fibrinogen gamma chain (Fig. 4, *A* and *B*, and Supplemental Fig. S11, yellow square).

Fifty four DEPs were downregulated in both stages II and III of the disease compared to stage I (Fig. 4, *A* and *B*, and S12, blue square). Haptoglobin was the protein with the highest number of DEPs (29 PFRs), followed by fibrinogen alpha chain (10 PFRs), alpha-2HS-glycoprotein (3 PFRs), and haptoglobin-related protein (2 PFRs). One DEP each was found for albumin, alpha-1-acid glycoprotein 1, angiogenin, apolipoprotein C-I, ceruloplasmin, clusterin, serotransferrin, and serum amyloid A-1 protein. Haptoglobin PFRs (Supplemental Fig. S12) perfectly overlapped with those characterized in C3 (described above). We again observed both C- and N-termini truncations in all 10 fibrinogen alpha chain PFRs at various positions. The average length of truncation was 58.8 amino acids. One fibrinogen alpha chain PFR was hydroxylated at a proline in position 565 (Supplemental Fig. S12).

**Fig. 5:**
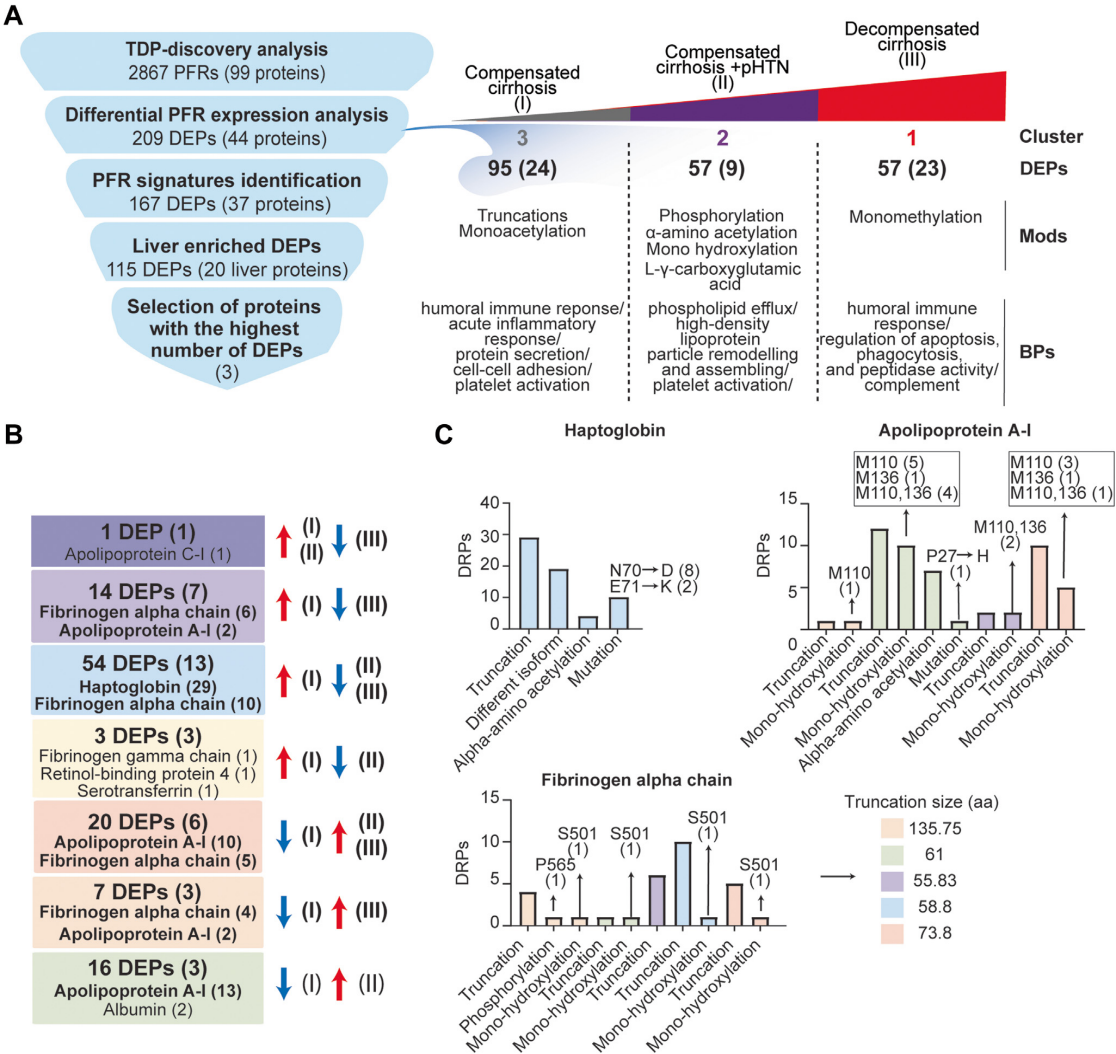
**Summary of quantitative TDP analysis of plasma proteoforms in patients with cirrhosis.***A*, strategy used to identify potential candidate proteoform biomarkers of liver cirrhosis and summary of the differential PFR expression analysis. Differentially expressed proteoforms (DEPs) were classified in clusters based on their upregulation in 1) compensated cirrhosis (stage I- cluster 3), 2) compensated cirrhosis with portal hypertension (stage II- cluster 2), and 3) decompensated cirrhosis (stage III- cluster 1). For each cluster, the number of upregulated DEPs with their relative proteins (in parenthesis), modifications, and biological processes are reported. *B*, composition of the identified liver-derived DEP signatures. The number of proteins from which the DEPs derive are shown in parenthesis. Proteins with the highest number of DEPs for each group are also reported and shown in bold. DEPs are grouped by color based on their upregulation or downregulation in disease stages as performed in Figure 4. For example, in the *red* group, 20 DEPs deriving from six proteins were upregulated in stages II and III of the disease as compared to stage I disease. Ten of these DEPs derived from apolipoprotein A-I and five derived from fibrinogen alpha chain. *C*, DEPs relative to haptoglobin, apolipoprotein A-I, and fibrinogen alpha chain, which are the three plasma proteins with the highest number of detected DEPs, are reported together with their modifications. Colors indicate the signature groups in *B* and *C*. PFR, proteoform; DEP, differentially expressed proteoform; Mod, modification; BP, biological process; aa, amino acids.

Twenty DEPs were upregulated in late stages (II and III) (Fig. 4, *A* and *B*, and S13, red square). These originated from apolipoprotein A-I (10 PFRs), fibrinogen alpha chain (5 PFRs), retinol-binding protein 4 (2 PFRs), apolipoprotein C-I (1 PFR), ceruloplasmin (1 PFR), and complement C4-A (1 PFRs). Sixteen DEPs were exclusively upregulated in stage II of the disease. The upregulated DEPs (Fig. 4, *A* and *B*, and S13, green square) were from apolipoprotein A-I (13 PFRs), albumin (2 PFRs), and fibrinogen alpha chain (1 PFR). Finally, seven DEPs were upregulated in stage III of the disease and included four PFRs derived from fibrinogen alpha chain, 2 from apolipoprotein A-I, and one from alpha-1 antitrypsin (Fig. 4, *A* and *B*, and S13, orange square). Collectively, 25 of the 27 apolipoprotein A-I PFRs that discriminated early stage (I) from later stage disease (II and III) were upregulated in later disease stages (Fig. 4 and Supplemental Fig. S13). These apolipoprotein A-I PFRs mostly contained N-terminus truncations at various locations and oxidation of methionine residues in positions 110 and 136. In addition, a proline to histidine point mutation occurred at position 27 in one PFR that was upregulated only in stage II disease (Supplemental Fig. S13, green square, PFR#5052090). Notably, all 25 of these PFRs were greater in length than those upregulated in stage I disease (Supplemental Fig. S11). Fibrinogen alpha chain PFRs upregulated in later-stage disease were all truncated at various residues at the N-terminus, but 8 of the 10 PFRs had a C-terminal truncation at residue 629. Finally, two fibrinogen alpha chain PFRs were hydroxylated at a proline in position 565 (Supplemental Fig. S13, green and red square), and one was hydroxylated at a serine in position 501 and had a phospho-proline at position 565 (Supplemental Fig. S13, orange square, PFR#7501917). The average length of truncation of fibrinogen alpha chain PFRs upregulated in later disease stages was higher than early-stage disease (stage III = 137.75, stages II and III = 73.8, stage II = 61, stage I = 55.83–58.8) (Fig. 5, Supplemental Figs. S11, and S12).

## Discussion

Using quantitative discovery TDP, we discovered DEPs in three stages of cirrhosis – compensated (stage I), compensated + pHTN (stage II), and decompensated (stage III) (Fig. 5). Our pilot discovery analysis identified 2867 PFRs in total, of which 663 were differentially expressed and 209 were analyzed in detail (Supplemental Table S2) after excluding hemoglobin proteoforms. Following the proteoform classification system (40), the percentage of proteins classified as level 1 increased significantly when PFRs considered as DEP were analyzed, rising from 47% to 82%. This increase was primarily due to the exclusion of hemoglobin, as only 21% of hemoglobin proteoforms were classified as level 1. Hemoglobin is well-known for having multiple variants due to genetic polymorphism and numerous PTMs (39) (https://www.uniprot.org/uniprotkb/P68871/entry#ptm_processing), which poses a challenge for their unambiguous assignment within a large set of candidate proteoforms in the database.

DEPs clustered into three groups based on their upregulation in the various stages of cirrhosis. Modifications to these DEPs differed depending on the stage of cirrhosis, and DEPs were involved in numerous, biologically relevant processes. One hundred sixty-seven DEPs discriminated stage I *versus* stages II and III, of which 115 derived from 20 proteins enriched in the liver at a transcriptional level. Proteoforms originating from fibrinogen alpha chain, apolipoprotein A-I, and haptoglobin were the most abundant and demonstrated varying degrees of modification across disease stages.

Apolipoprotein A-I is a lipoprotein produced by both the liver and intestine and is a key component of high-density lipoprotein (HDL). Its primary function is in reverse cholesterol transport, but it is also an important mediator of immunological and inflammatory processes (41). Structural changes, mutations, PTMs, and decreased synthesis of apolipoprotein A-I have been associated with multiple disease entities (42). In the context of liver disease, decreased apolipoprotein A-I levels have been observed in patients with cirrhosis (43, 44, 45, 46, 47). Although apolipoprotein A-I is a component of an established screening tool for liver fibrosis called the “Fibrotest,” scarce studies have assessed the ability of apolipoprotein A-I levels to predict decompensation events or death (45, 46, 47). In the most recent of these, Gurbuz *et al* used bottom-up proteomics to analyze the serum proteome of hepatocellular proteins in patients with cirrhosis *versus* healthy controls. Of 903 proteins identified, 81 proteins of hepatocellular origin were differentially expressed between groups: 65 downregulated and 16 upregulated in subjects with cirrhosis. Decreased serum levels of apolipoprotein A-I were observed in cirrhosis patients and correlated with a transition from stable to unstable decompensated cirrhosis (47). In contrast, we observed most apolipoprotein A-I proteoforms were upregulated in later stage disease. Modifications of apolipoprotein A-I proteoforms included truncation, oxidation of two methionine residues, and alpha-amino acetylation. Nearly all the observed proteoforms in later-stage disease (Fig. S12) contained more amino acids than proteoforms seen in stage I disease. However, most of these proteoforms were either truncated at their N- or C-termini. Additionally, some were partially processed or in their unprocessed N-terminal forms. This may be due to the secretion of apolipoprotein A-I’s prepropeptide and/or inappropriate cleavage of the prepropeptide in subjects with more severe diseases (42, 48), ultimately leading to dysfunction of apolipoprotein A-I proteins. In general, the N- and C-terminal domains of apolipoprotein A-I are typically associated with lipid-binding activities (49, 50, 51). Alterations in these domains, such as truncations or unprocessed N-terminal forms of apolipoprotein A-I, might be linked loss-of-function in HDL-cholesterol binding. The hydroxylation of apolipoprotein A-I may exacerbate this as oxidation of HDL can negatively affect cholesterol efflux, anti-apoptotic effects, anti-peroxidation, and other anti-inflammatory actions (52). More specifically, oxidation of methionine residues within the apolipoprotein A-I protein can interfere with apolipoprotein A-I’s interaction with lecithin cholesterol acyltransferase, impairing reverse cholesterol transport (53).

Haptoglobin, an acute-phase protein that scavenges cell-free hemoglobin, has also been studied as a biomarker of liver disease severity. Like apolipoprotein A-I, decreased haptoglobin levels are correlated with higher degrees of liver fibrosis (54, 55). There is also evidence to suggest that glycoforms of haptoglobin may be useful in differentiating various stages and etiologies of liver disease (55, 56, 57, 58, 59, 60, 61). However, little is known about the specific effect of cirrhosis progression on haptoglobin function, and there are few to no studies that have investigated haptoglobin and its modifications as a predictor of future decompensation events. We observed DEPs of haptoglobin were relatively upregulated in stage I disease (Figs. 3, 4, and Supplemental Fig. S12). All haptoglobin DEPs were identified as a truncation, but review of the amino acid sequences would suggest DEPs derive from mature alpha-1 or alpha-2 haptoglobin chains (www.uniprot.org, (62)). This is additionally supported by the presence of two point mutations at residues 71 and 72, which are known mutations of two alleles of the alpha-1 chain, *alpha-1F* and *alpha-1S* (62). Interestingly, the alpha-amino acetylation PTM observed in our study was present only on the alpha-1 chain and has not been previously studied in the context of cirrhosis. Alpha-amino acetylation can impact protein half-life, protein folding, protein-protein interactions, and membrane targeting, but it is unknown what effect this specific PTM has on haptoglobin. As such, future investigations should elucidate the importance of this PTM in the setting of cirrhosis as it may impact the production of and function of mature haptoglobin proteins in patients with more severe diseases.

Cirrhosis-associated coagulopathy has been well described, and subjects with cirrhosis are predisposed to both bleeding and thrombotic events (63). Patients with stable liver disease generally have normal levels of fibrinogen, but as disease progresses, fibrinogen levels steadily decrease (64, 65). Dysfibrinogenemia also plays an important role in coagulopathy as modifications to fibrinogen subunits can impact both structure and function (66, 67). Truncated forms of fibrinogen can influence blood clotting and stability (68, 69). In our cohort, fibrinogen DEPs originated most commonly from the alpha chain and were of varying lengths (S11-S13). Given the full-length fibrinogen alpha chain is ∼610 amino acids after translation and intracellular processing (70), it is likely these alpha chain DEPs are byproducts of fibrin breakdown based upon their much shorter amino acid sequences (Supplemental Fig. S9). The discrepancies in the length of detected DEPs across clusters may be a consequence of complex alterations in fibrin degradation and of the proteinases important for fibrin breakdown (*i.e.*, thrombin, plasmin, neutrophil elastase, and proteinase 3) in various stages of disease. This is consistent with studies that have shown D-dimer, a fibrin degradation product, is elevated in patients with cirrhosis and is associated with increased mortality in patients with later stage disease (71, 72, 73, 74, 75). Further characterization of these specific DEPs can provide a starting point for mechanistic investigations of fibrinolysis in patients with cirrhosis and may present an opportunity for therapeutic intervention, whether that be on the fibrinogen molecule itself or on the proteinases responsible for its degradation.

While focused primarily on cases with the highest number of DEPs like apolipoprotein A-I, haptoglobin, and fibrinogen alpha, several other liver proteins with fewer DEPs could still help distinguish different stages of cirrhosis. Some of those DEPs belong to proteins that were already associated with liver cirrhosis, such as apolipoprotein C-I and the retinol binding protein 4. Apolipoprotein C-I is a protein-regulating lipid transport and metabolism associated with lipid disorders, inflammation, and immunity (76). In our study, we found four differentially expressed forms of apolipoprotein C-I. Previous studies conducted on the serum of patients with fibrosis, cirrhosis, and hepatocellular carcinoma indicated that the mature, secreted form of apolipoprotein C-I lacking the 26-residue signal peptide is downregulated in these patients (77), concordant with the results reported here. Indeed, we found three apolipoprotein C-I proteoforms downregulated in stage III. Among those, one represents the secreted form (residues 27–83) that is also amino acetylated. This proteoform is downregulated in stage III and upregulated in stage I. The other two DEPs are truncations of the mature apolipoprotein C-I lacking two residues at the N-terminus (residues 29–83), previously found downregulated in the colorectal cancer patients (78). In our case, those DEPs differ only for the presence/absence of amino acetylation, with the modified proteoform being downregulated both in stage II and III and the unmodified form downregulated in stage III and upregulated in stages I and II. We also found one apolipoprotein C-I proteoform upregulated in the late disease stages (II-III). Unexpectedly, this proteoform (residue 13–83) maintains part of the signal peptide, indicating that in the late stages of the disease, there may be deficit in apolipoprotein C-I processing into its mature form.

Another protein of interest is retinol-binding protein 4 (RBP4), which is an adipocytokine produced in the liver that transports the retinol (vitamin A) in the blood (79). It was previously shown that in chronic liver disease patients, RBP4 is lower than those in healthy individuals and its levels correlate with the severity of the disease (80, 81, 82, 83). In addition, it is considered as a promising diagnostic and prognostic marker of hepatocellular carcinoma (84). Multiple RBP4 variants were identified previously, including the loss of one or two C-terminal leucine residues that are involved in kidney disease (85). Our TDP analysis found three DEPs for RBP4. One of these is a truncated proteoform consisting of residues 19 to 194 of the protein with a mutation in position 73 (A to T) and a methylation on R139 upregulated in stage I and downregulated in stage II of the disease. The mutation in position 73 is associated with congenital eye disease and it was reported to decrease RBP4 binding to retinol and reduce retinol levels in the serum, which are reduced in cirrhosis (86, 87). The other two PFRs include residues 19 to 201 and are upregulated in late stages of the disease. Also in this case, we observed that late stages of the disease are associated with a reduced protein truncation, a phenomenon known to occur in liver cirrhosis due to reduced liver function that affects protein processing (88). Functional studies must follow to determine the significance of these DEP in disease progression.

There are key limitations to our study. First, we are limited by the sample size of patients as this was an early pilot study to identify biomarker candidates. Considering this, a benefit of our proteoform-based analysis is the degree of resolution achieved, which can identify significant differences even in a small cohort study. Still, targeted validation studies in a larger cohort are needed to confirm and expand upon our findings. Second, our cohort consisted mostly of patients with alcohol or MASH-related cirrhosis, as they are the two most common etiologies of cirrhosis in the United States. Future studies will need to include additional patients with other etiologies of disease to ensure generalizability across cirrhosis populations. PEPPI-MS workflow enabled the identification of less-abundant proteoforms and deeper proteoform profiling in plasma. However, challenges in top-down MS analysis of high molecular weight proteins (MW > 30 kDa) persist due to lower signal-to-noise ratio from sample loss, signal decay, and poor separation in complex samples (31, 89, 90). Recent advancements in TDP have significantly addressed such limitations. For example, the Ge group developed a 2D separation platform using serial size exclusion chromatography coupled with RPLC to detect proteoforms up to 223 kDa in human heart tissue (35, 91). Shen et al. demonstrated the benefits of capillary zone electrophoresis-MS for large-scale TDP (92), achieving plasma proteoform characterization in the 3 to 70 kDa range. Our group utilized a modified Proteograph workflow to analyze human plasma, identifying over 2800 proteoforms (93). New TDP acquisition methods, such as LC-FAIMS-MS2 (32), have further reduced spectral complexity and increased the depth of proteoform profiling. Additionally, techniques like proton transfer charge reduction (94) and individual ion mass spectrometry have enhanced MS1 and MS/MS analyses of large proteoforms and their fragment ions (95, 96), applicable to plasma proteome studies. Finally, it is possible some proteoforms identified in our discovery TDP analysis are a result of electrospray ionization or sample handling during preparation. Specifically, oxidation is a known byproduct of such techniques, and we can assume that at least a few of the proteoforms detected may be a result of sample processing. However, the fact we observed significant differences in the expression of a number of oxidized proteoforms across samples that underwent identical processing makes this less likely and suggests BP are driving these differences.

In summary, quantitative TDP of plasma proteins from patients with cirrhosis revealed unique plasma proteoform profiles associated with stages of disease in this pilot study. To our knowledge, this is the first report of such proteoform analysis in patients with cirrhosis. Although we focused on the most abundant DEPs (apolipoprotein A-I, haptoglobin, and fibrinogen alpha chain), there were other PFRs identified in this study that may be candidate biomarkers for disease progression, both individually and in combination with other PFRs. Moving forward, we will perform targeted studies on larger cohorts to validate and further characterize the identified PFRs. The TDP methodology employed here could greatly increase the accuracy of blood-based analyses due to the identification of DEPs that are highly sensitive and specific probes of the underlying biology over time. In a clinical setting, understanding the change in PFRs from one stage of cirrhosis to the next would enable prompt recognition of patients transitioning from compensated to later-stage disease, presenting an opportunity for targeted risk reduction or early intervention in such patients.

## Data availability

Raw files and tdReport files can be found in Massive (Accession MSV000094311, with some examples of MS/MS spectra can be found in Figs. S14-S16. The search results in the tdReport format can be viewed by using TDViewer freely available at http://topdownviewer.northwestern.edu. The search results were further analyzed, and figures were generated with R-custom scripts that are available on request.

## Supplemental data

This article contains supplemental data.

## Conflicts of interest

N. L. K. is involved in entrepreneurial activities in Top-down proteomics and consults for Thermo Fisher Scientific. R. D. M. is a current Thermo Fisher Scientific employee. The other authors have declared that they have no conflict of interest with the contents of this article.
