# Supplementary material for: Top-Down Proteomics Identifies Plasma Proteoform Signatures of Liver Cirrhosis Progression
Source: Mol Cell Proteomics. 2024 Nov 8;23(12):100876. doi: 10.1016/j.mcpro.2024.100876 (PMC11664408; doi:10.1016/j.mcpro.2024.100876)
Supplement: Supplemental data [file mmc4.docx]

**Top-Down Proteomics Identifies Plasma Proteoform Signatures of Liver**

**Cirrhosis Progression**

Eleonora Forte, Jes M. Sanders, Indira Pla, Vijaya Lakshmi Kanchustambham, Michael A. R. Hollas, Che-Fan Huang, Aniel Sanchez, Katrina N. Peterson, Rafael D. Melani, Alexander Huang, Praneet Polineni, Julianna M. Doll, Zachary Dietch, Neil L. Kelleher, and Daniela P. Ladner.

**Contents**

[Fig. S1. Clinical parameters of patients enrolled in this study 2](#_Toc183287981)

[Fig. S2. T Common proteoform modifications detected in the TDP analysis 4](#_Toc183287982)

[Fig. S3. Monoisotopic Mass Distribution. 5](#_Toc183287983)

[Fig. S4. Differentially expressed proteoforms (DEPs) from decompensated (III) vs compensated with portal hypertension (+ pHTN) (II) patients 6](#_Toc183287984)

[Fig. S5. Differentially expressed proteoforms (DEPs) from decompensated (III) vs compensated (I) patients 7](#_Toc183287985)

[Fig. S6. Differentially expressed proteoforms (DEPs) from compensated with portal hypertension (+ pHTN) (II) vs compensated (I) patients 8](#_Toc183287986)

[Fig. S7. Proteoform modifications in pairwise comparisons of cirrhosis stages 9](#_Toc183287987)

[Fig. S8. Proteoform modifications compared across clusters. 10](#_Toc183287988)

[Fig. S10. Heatmaps of quantified proteoforms of proteins not enriched in the liver at transcriptional level 12](#_Toc183287989)

[Fig. S11. Individual proteoforms upregulated in early-stage cirrhosis 13](#_Toc183287990)

[Fig. S12. Individual proteoforms upregulated in Stage I disease. 14](#_Toc183287991)

[Fig. S13. Individual proteoforms upregulated in late-stage cirrhosis 15](#_Toc183287992)

[Fig. S14. MS1 and Tandem-MS/MS Fragmentation spectra of Apolipoprotein C-I 16](#_Toc183287993)

[Fig. S15. MS1 and Tandem-MS/MS Fragmentation spectra of Haptoglobin 17](#_Toc183287994)

[Fig. S16. MS1 and Tandem-MS/MS Fragmentation spectra of Haptoglobin 18](#_Toc183287995)


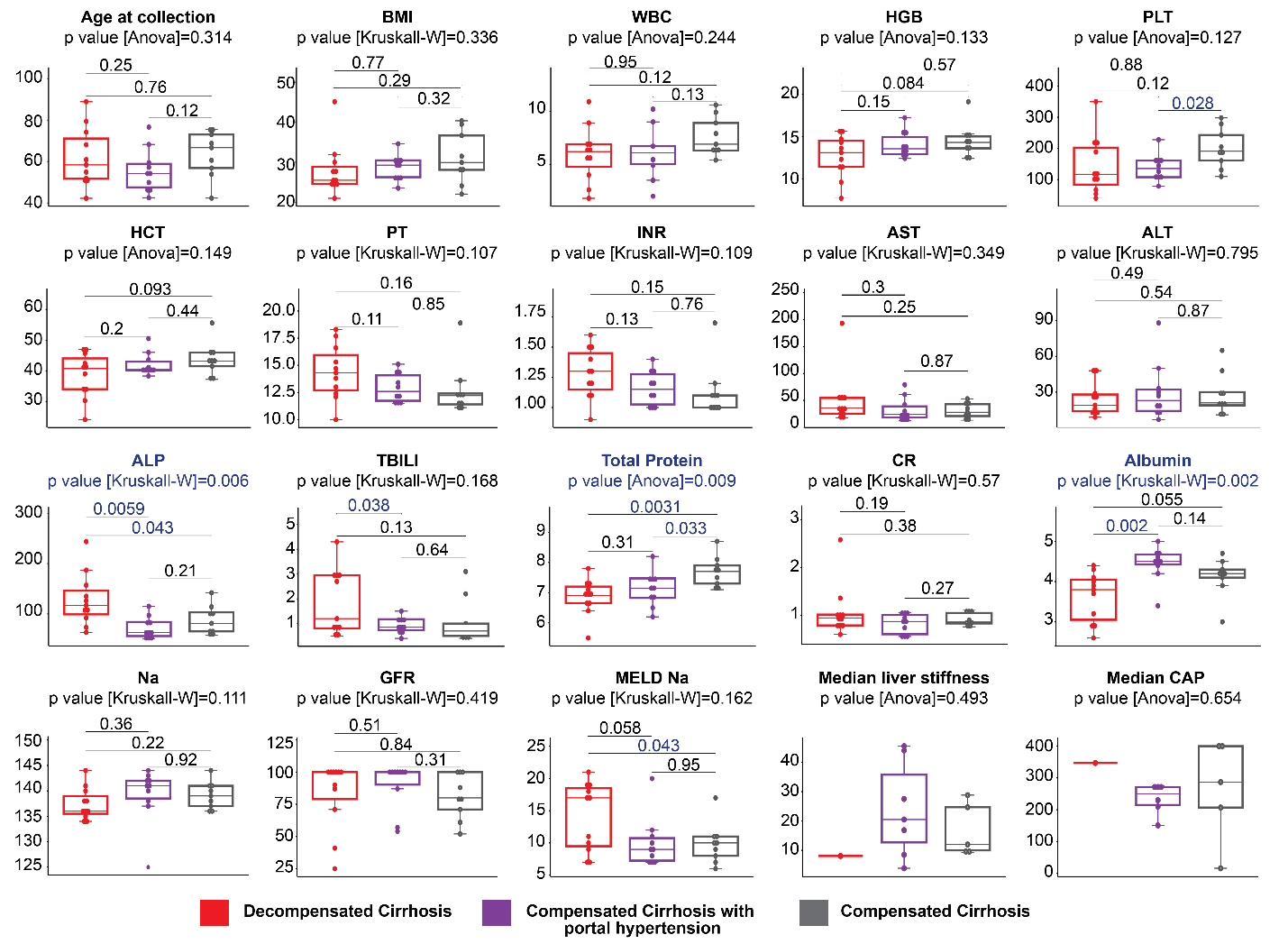


Fig. S1. Clinical parameters of patients enrolled in this study. Clinical parameters shown in blue are statistically significant different among groups. Significance (p value <0.05) was calculated by Anova or Kruskall-Wallis (Kruskall-W) tests, depending on the data distribution determined by Shapiro test. Abbreviations: BMI (body mass index), WBC (white blood cell count), HGB (hemoglobin), PLT (platelets), HCT (hematocrit), PT (prothrombin time), INR (international normalized ratio), AST (aspartate aminotransferase), ALT (alanine aminotransferase), ALP (alkaline phosphatase), TBILI (total bilirubin), CR (serum creatinine), Na (Sodium), GFR (glomerular filtration rate), MELD-Na (Model for End Stage Liver Disease Sodium), CAP (controlled attenuation parameter).


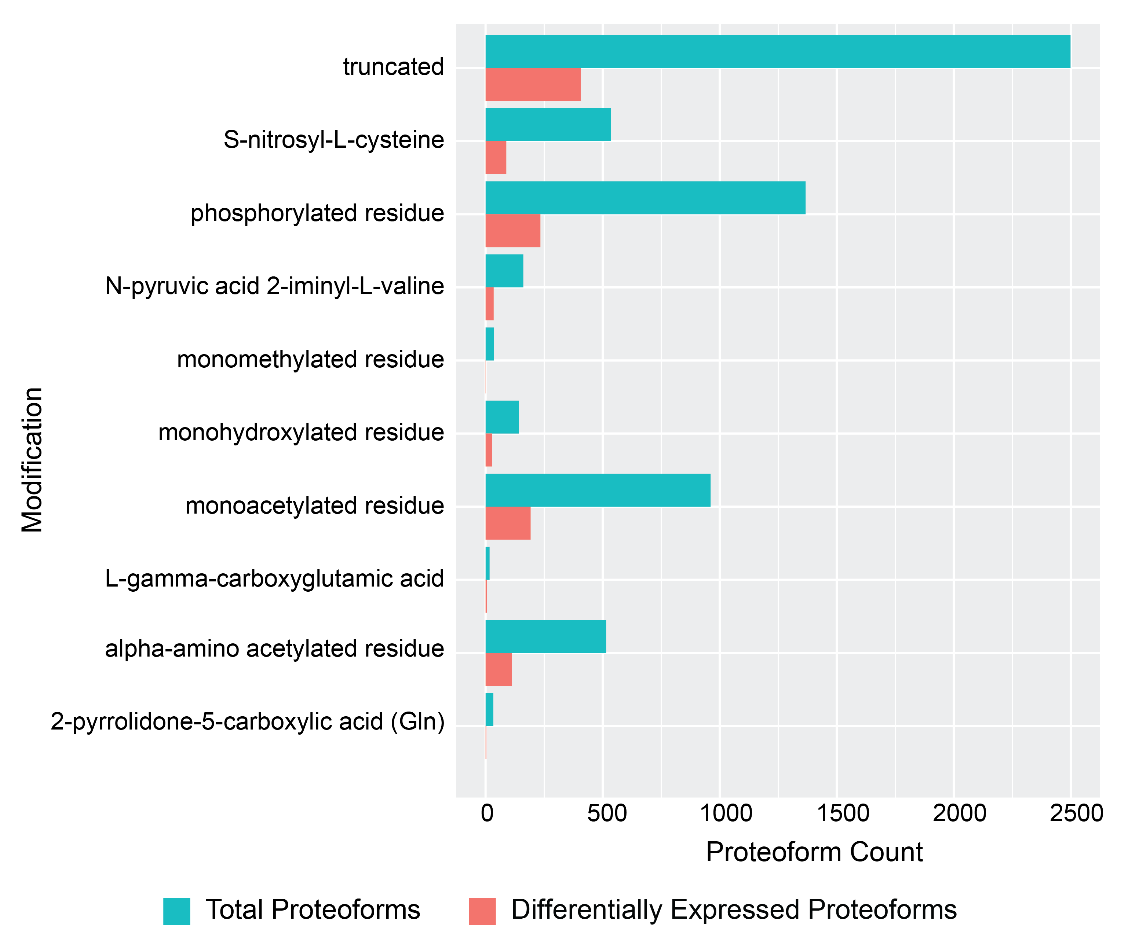


Fig. S2. T Common proteoform modifications detected in the TDP analysis**.** The number of truncations and top-10 most common post-translational modifications identified in the total and differentially expressed proteoforms (DEPs) captured in the discovery LC-MS/MS TDP analysis.

**
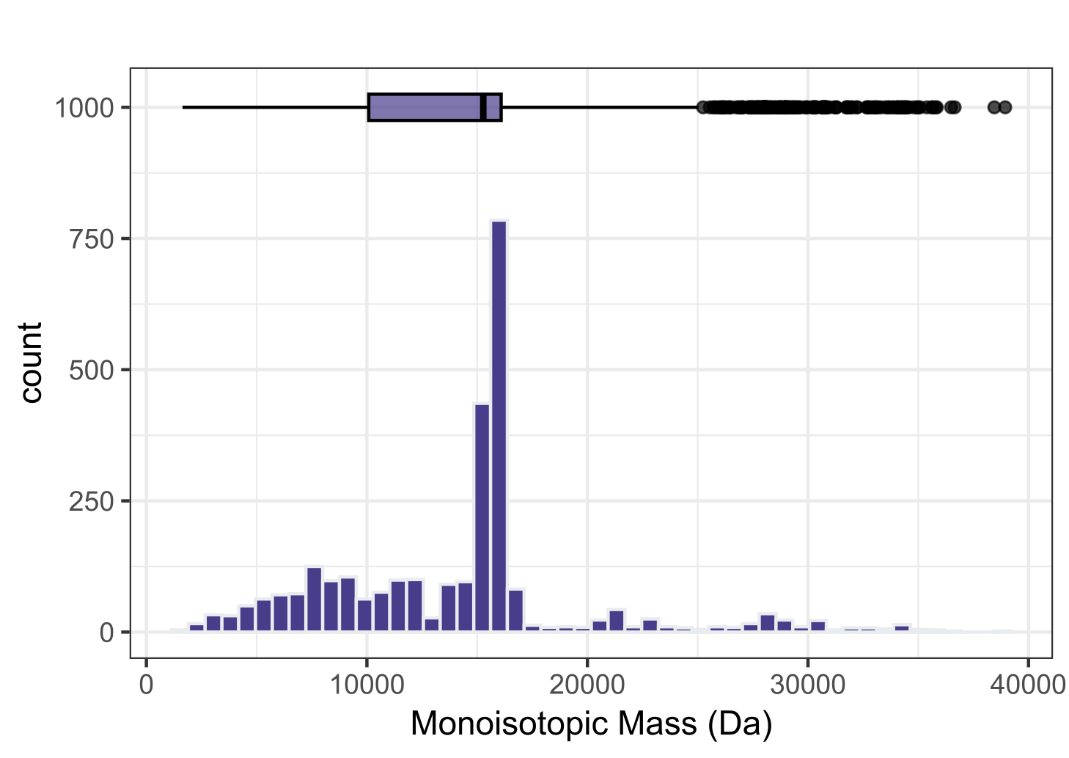
**

# Fig. S3. Monoisotopic Mass Distribution.


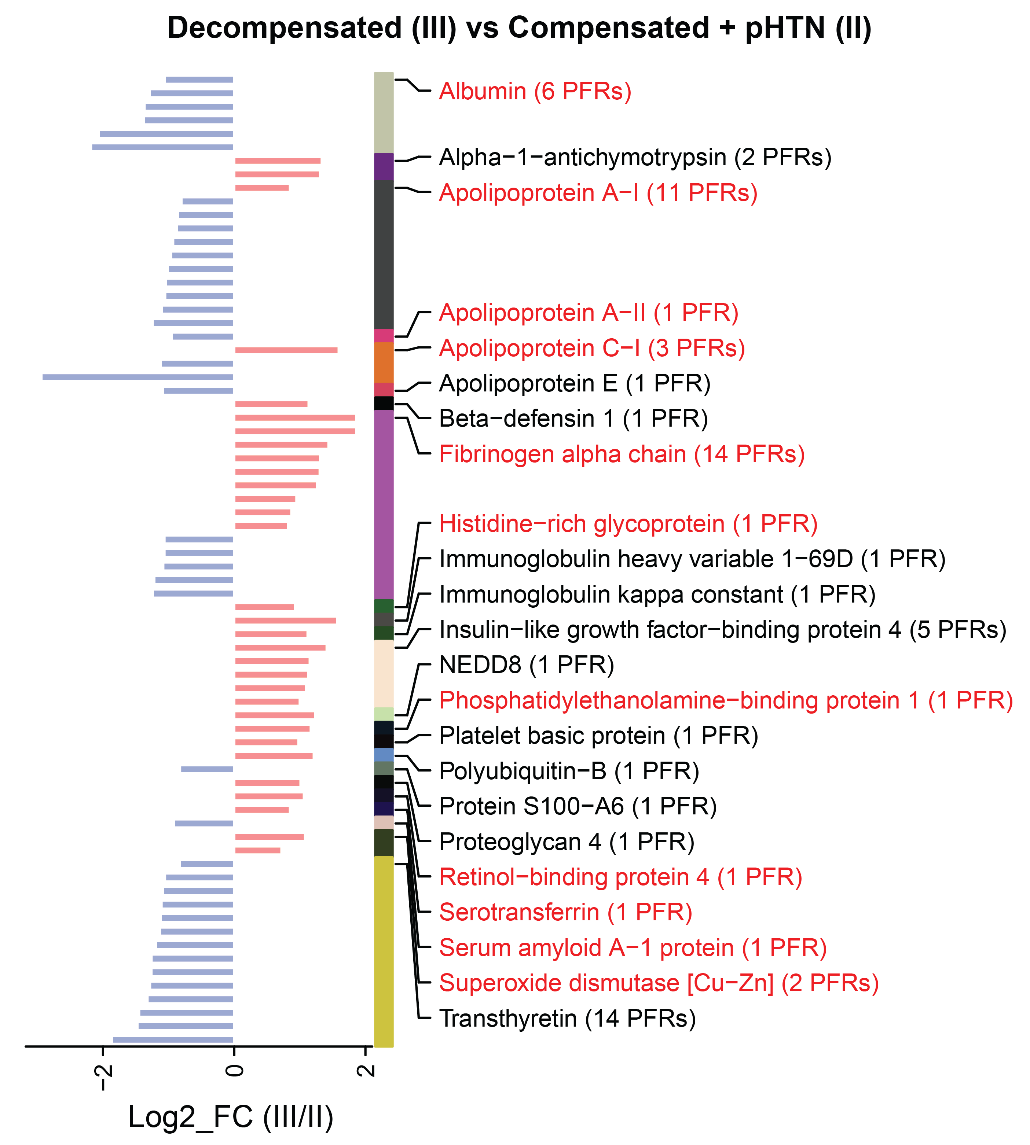


Fig. S4. Differentially expressed proteoforms (DEPs) from decompensated (III) vs compensated with portal hypertension (+ pHTN) (II) patients. DEPs are grouped by proteins, and then ordered by fold change. The protein and number of DEPs derived from that protein are shown to figure right. For example, fibrinogen alpha chain had 14 DEPs identified. Proteins highlighted in red are enriched in liver at a transcriptional level. Abbreviations: PFR (proteoform).

**
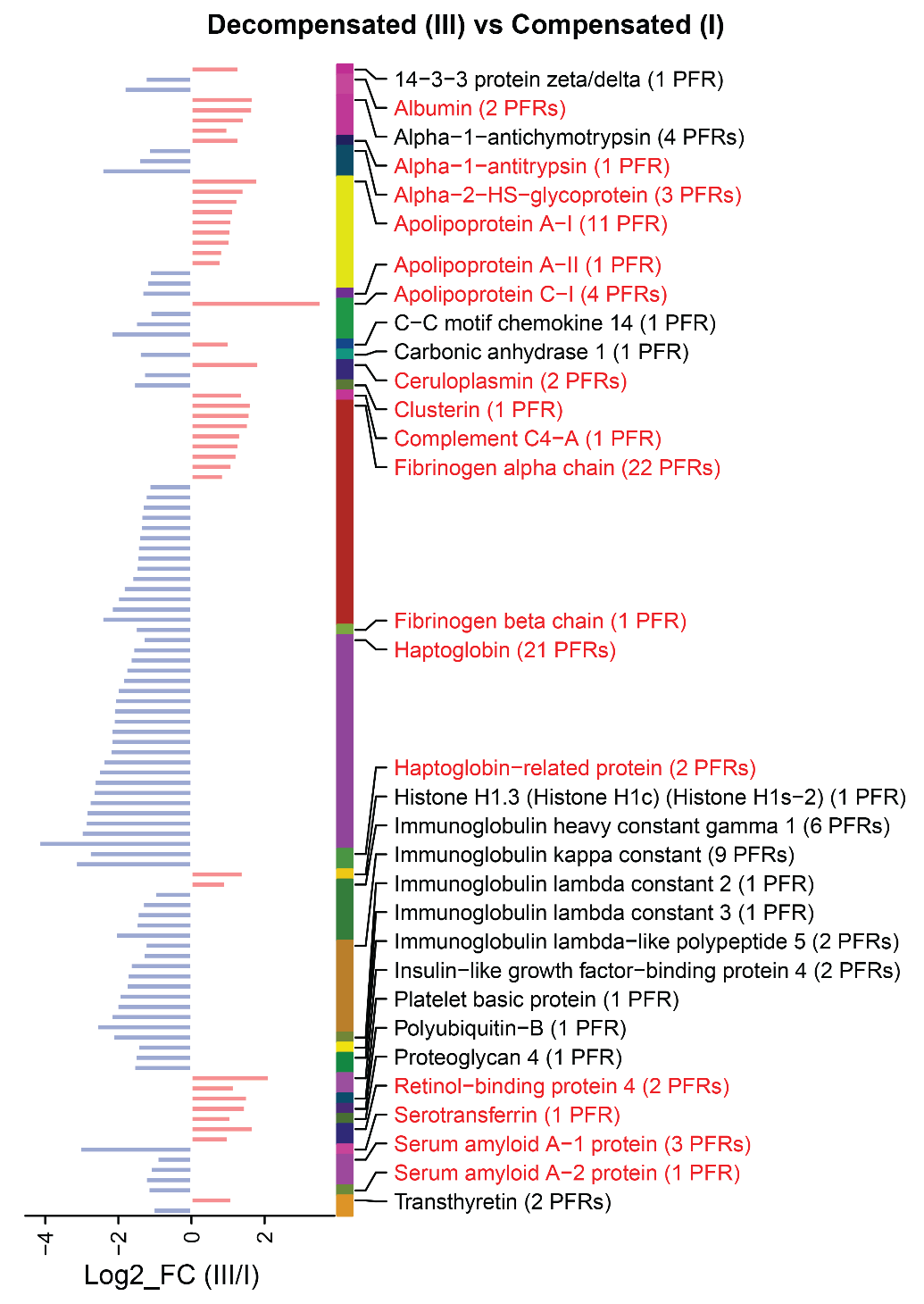
**

Fig. S5. Differentially expressed proteoforms (DEPs) from decompensated (III) vs compensated (I) patients. DEPs are grouped by proteins, and then ordered by fold change. The protein and number of DEPs derived from that protein are shown to figure right. For example, haptoglobin related protein had 2 DEPs identified. Proteins highlighted in red are enriched in liver at a transcriptional level. Abbreviations: PFR (proteoform).

**
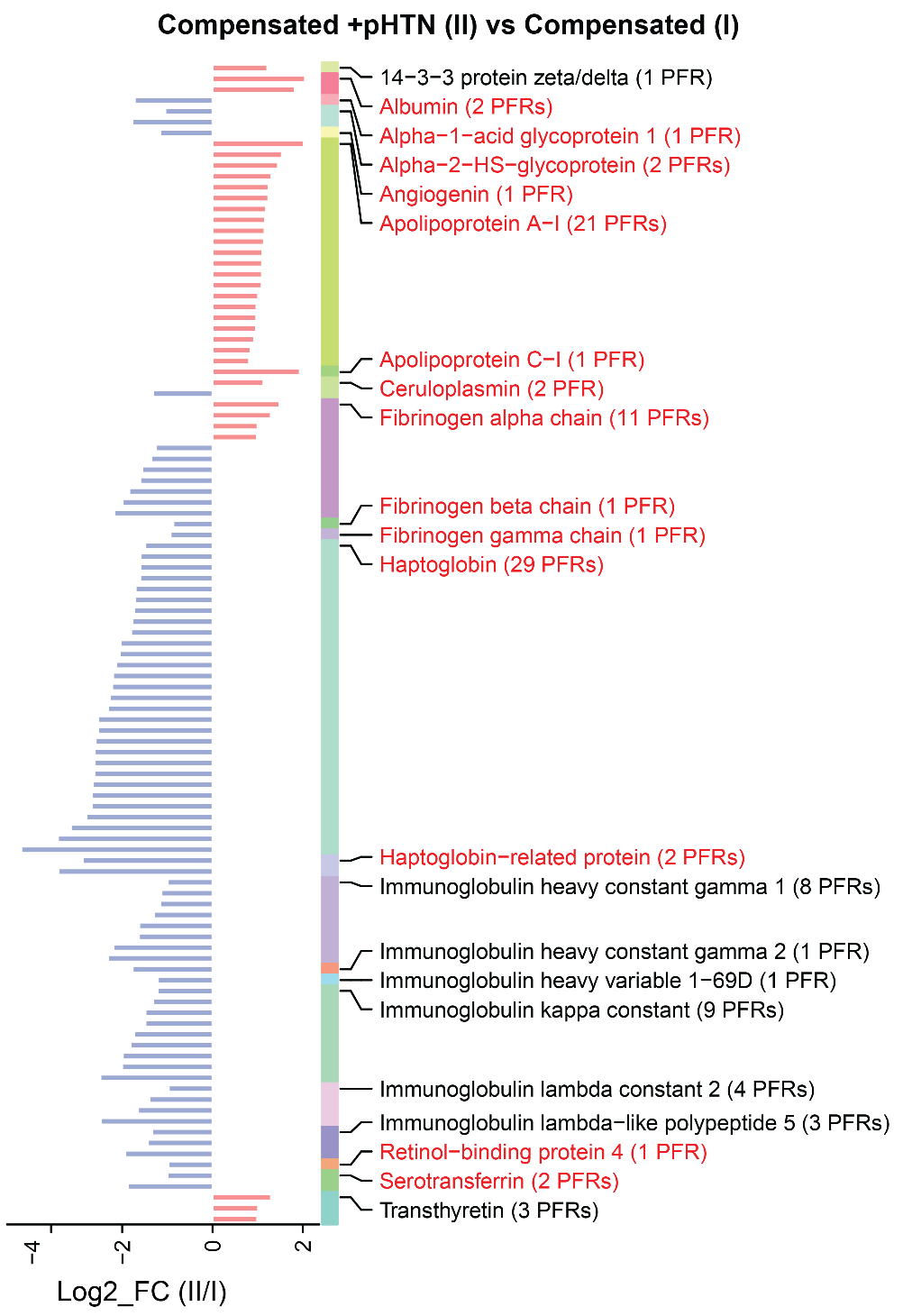
**

Fig. S6. Differentially expressed proteoforms (DEPs) from compensated with portal hypertension (+ pHTN) (II) vs compensated (I) patients. DEPs are grouped by proteins, and then ordered by fold change. The protein and number of DEPs derived from that protein are shown to figure right. For example, haptoglobin related protein had 2 DEPs identified. Proteins highlighted in red are enriched in liver at a transcriptional level. Abbreviations: PFR (proteoform).

**
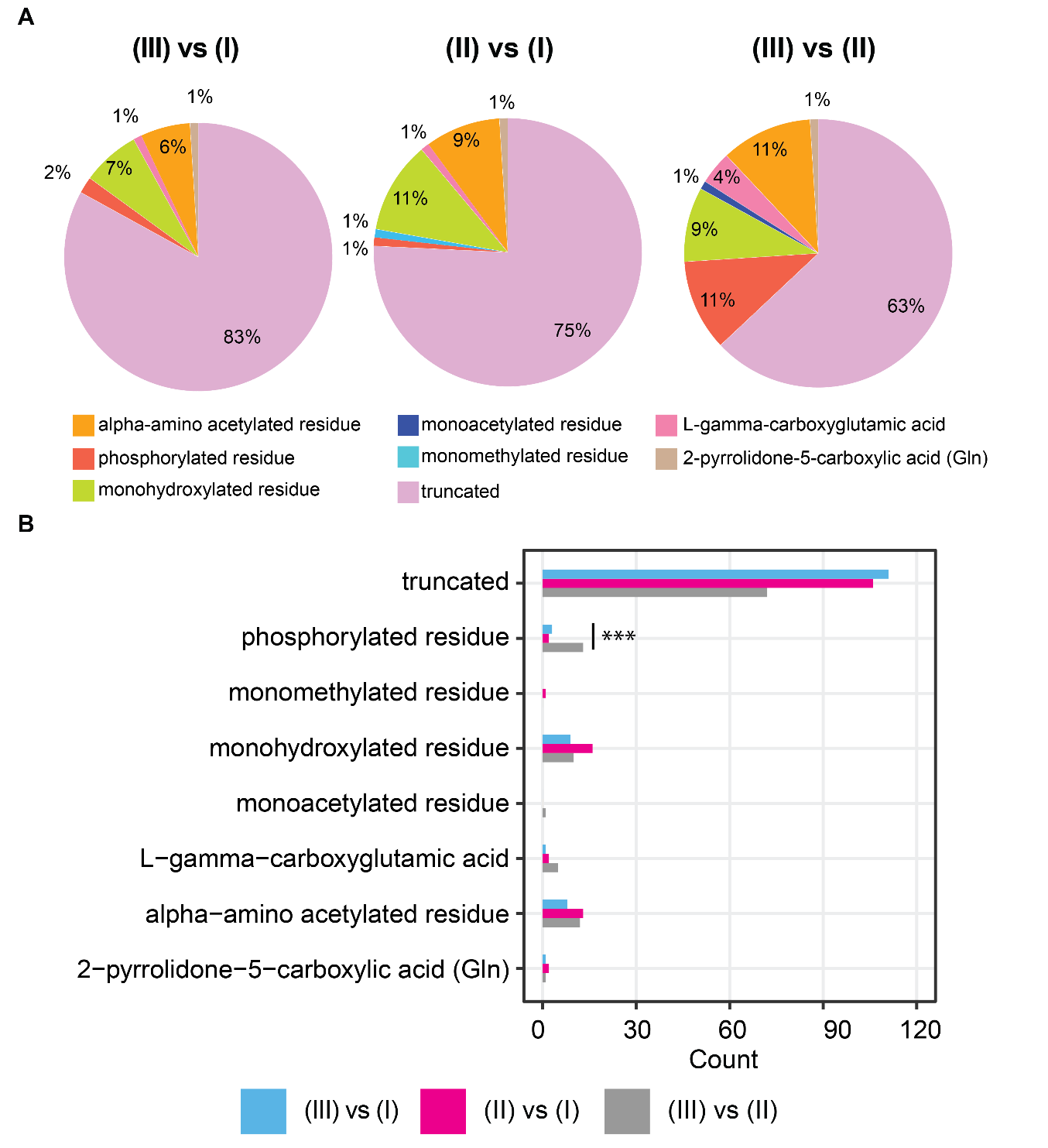
**

Fig. S7. Proteoform modifications in pairwise comparisons of cirrhosis stages**. A)** Percentages and **B)** absolute number of differentially expressed proteoform (DEP) modifications identified in pairwise comparisons of Stage III vs I, II vs I, and III vs II. For example, 11% of proteoforms differentially expressed between Stages III and II were phosphorylated residues (red portion of pie chart). Statistical significance was calculated with the Fisher exact test (adj. *p*-values: ***<0.001, **<0.01, *<0.05).

**
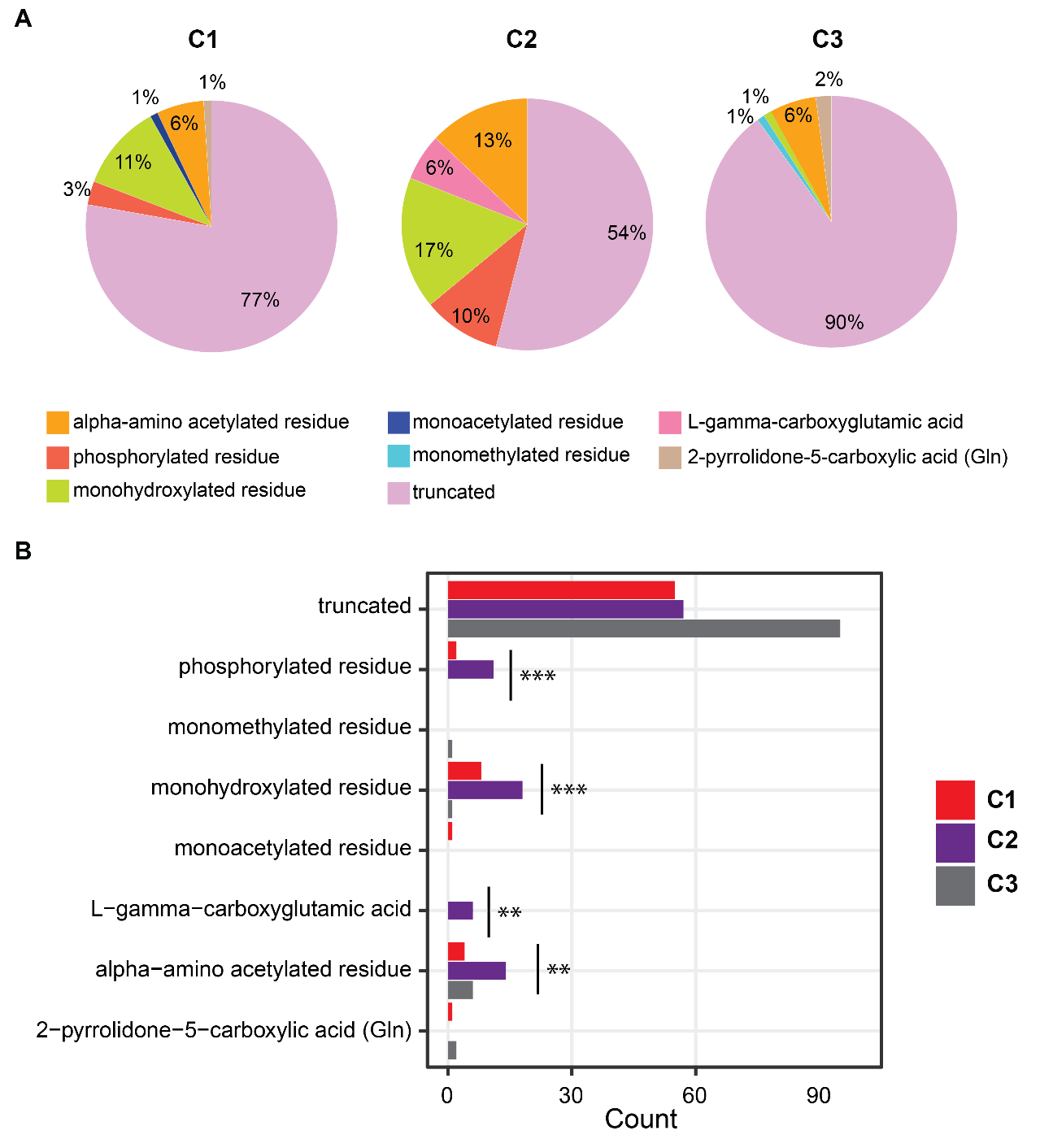
**

Fig. S8. Proteoform modifications compared across clusters. **A)** Percentages and **B)** Absolute number of differentially expressed proteoform (DEP) modifications identified in clusters 1 (C1), 2 (C2), and C3. For example, 90% of modifications to DEPs that clustered into C3 were identified as truncations (light purple portion of the pie chart). Statistical significance was calculated with the Fisher exact test (adj. *p*-values: ***<0.001, **<0.01, *<0.05).

**
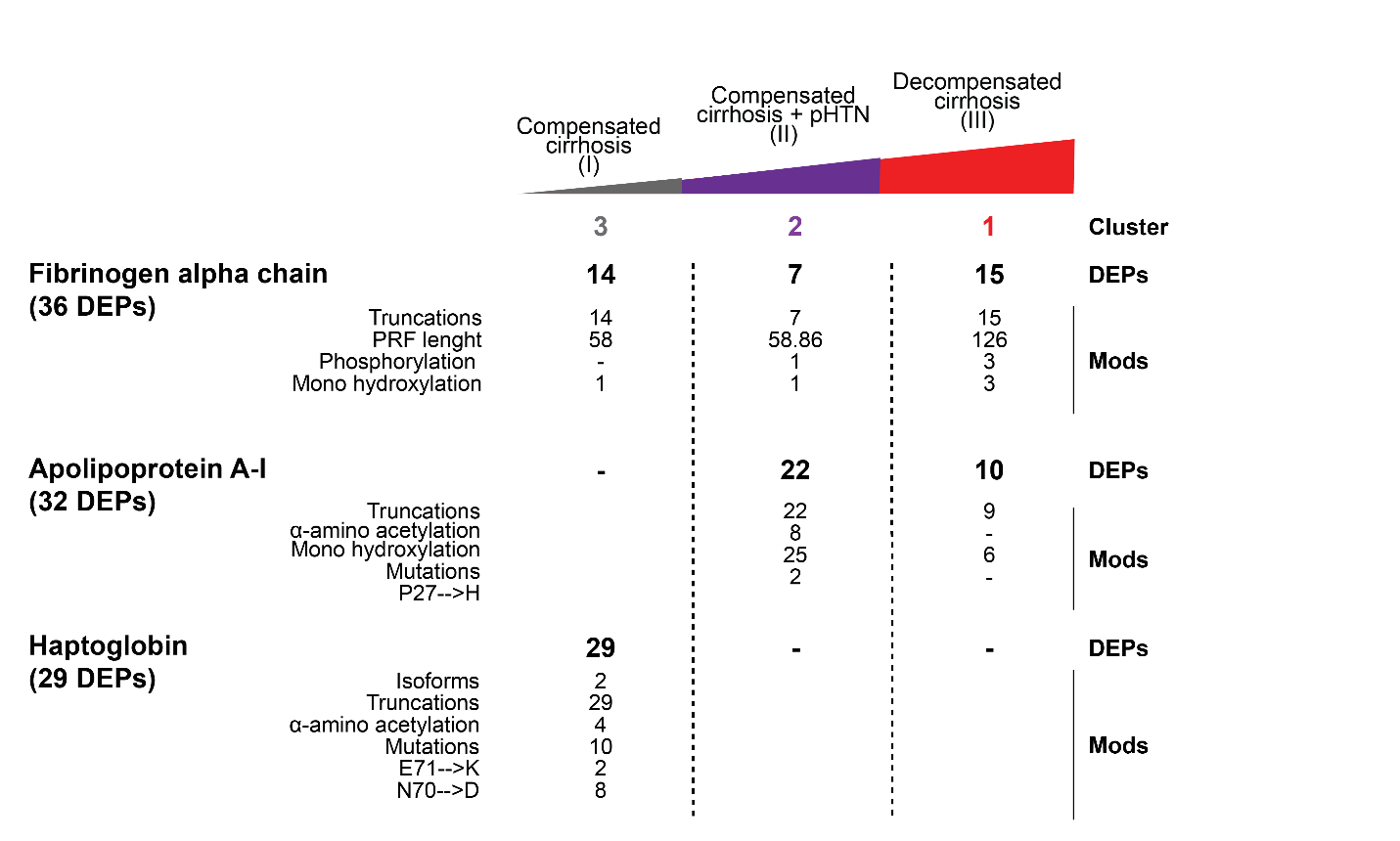
**Fig. S9. Most common, liver-enriched differentially expressed proteoforms (DEPs) identified in TDP analysis**.** DEPs relative to Fibrinogen alpha chain, Apolipoprotein A-I, and Haptoglobin are reported together with their modifications in the 3 clusters established in **Fig. 2**. Proteins are shown with the number of DEPs in parentheses. Abbreviations: DEP (Differentially expressed proteoform), Mod (Modification).


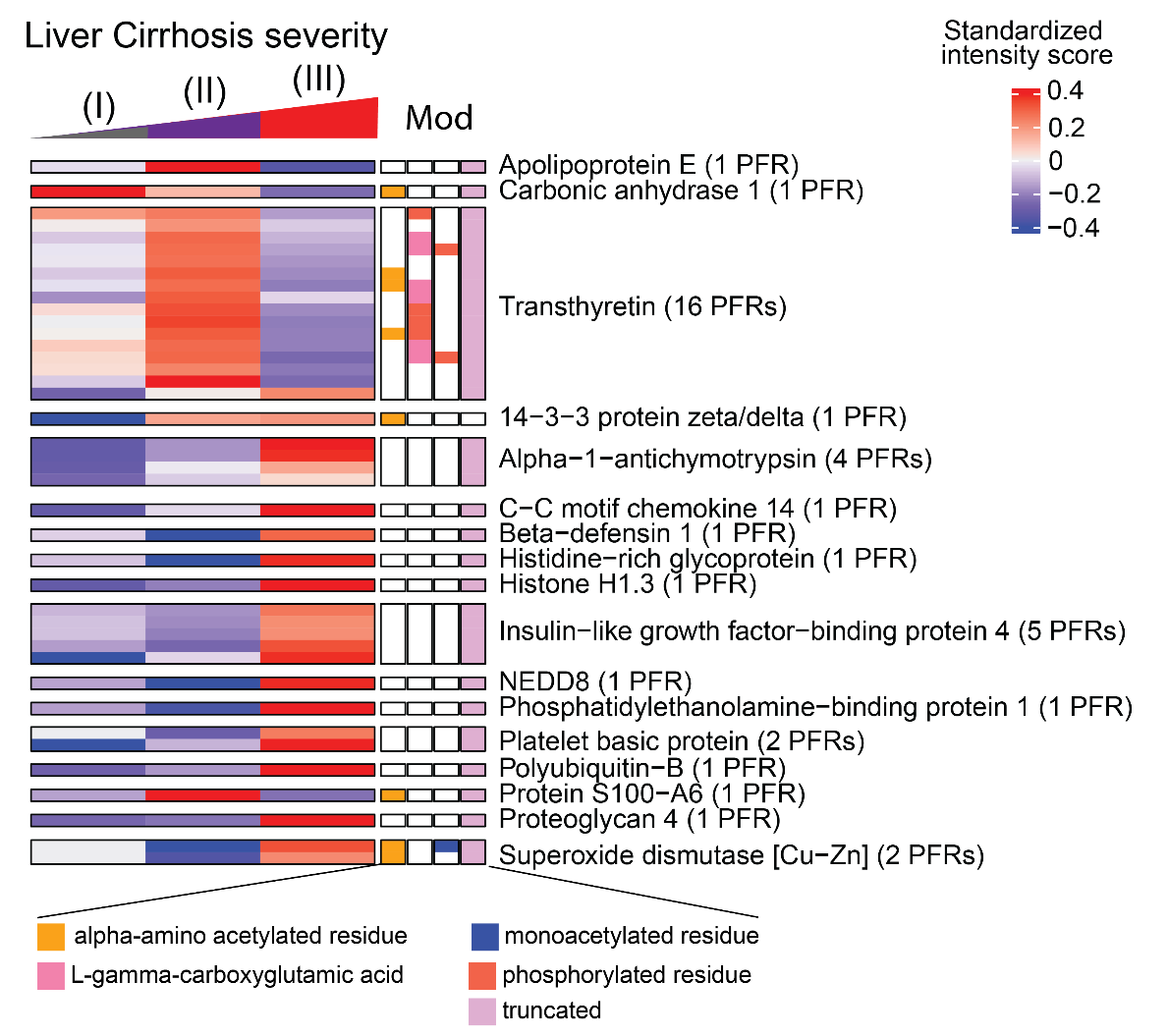


Fig. S10. Heatmaps of quantified proteoforms of proteins not enriched in the liver at transcriptional level**.** Associated proteoform modifications are shown and defined by the figure legend. The proteins and number of identified proteoforms derived from each protein are shown to figure right. Abbreviations: PFR (proteoform), Mod (modification).


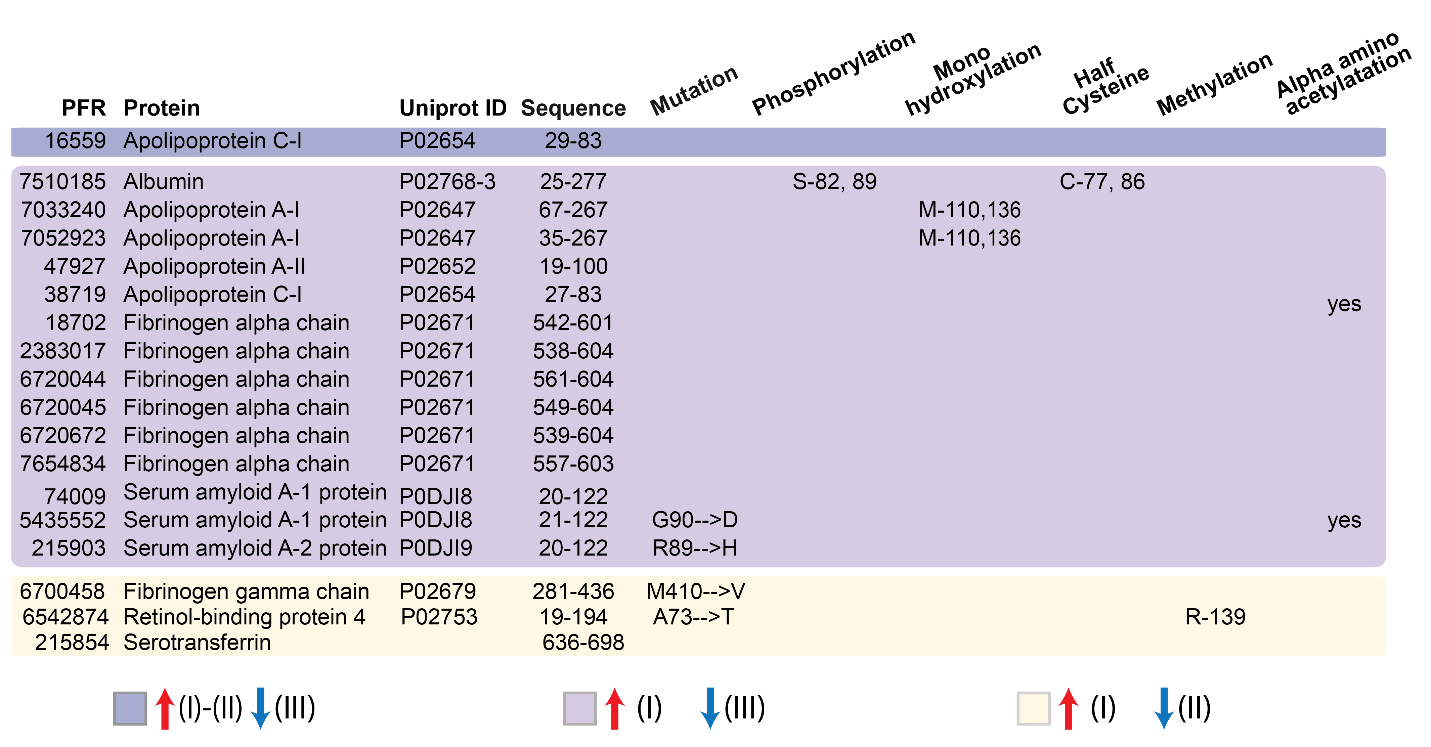


Fig. S11. Individual proteoforms upregulated in early-stage cirrhosis**.** Differentially expressed proteoforms (DEPs) significantly upregulated in stages (I) and (II) and downregulated in stage (III) (dark purple), upregulated in stage (I) and downregulated in stage (III) (light purple), upregulated in stage (I) and downregulated in stage (II) (yellow)**.** Each proteoform is shown with its unique proteoform number, protein name,Uniprot ID, amino acid sequence relative to the Uniprot ID, any relevant mutations, and presence or absence of post-translational modifications. Colors are based on the proteoform signatures created in **Fig. 4**. Abbreviations:  PFR (proteoform).

*Note that half Cysteine could be artifact during the identification process.


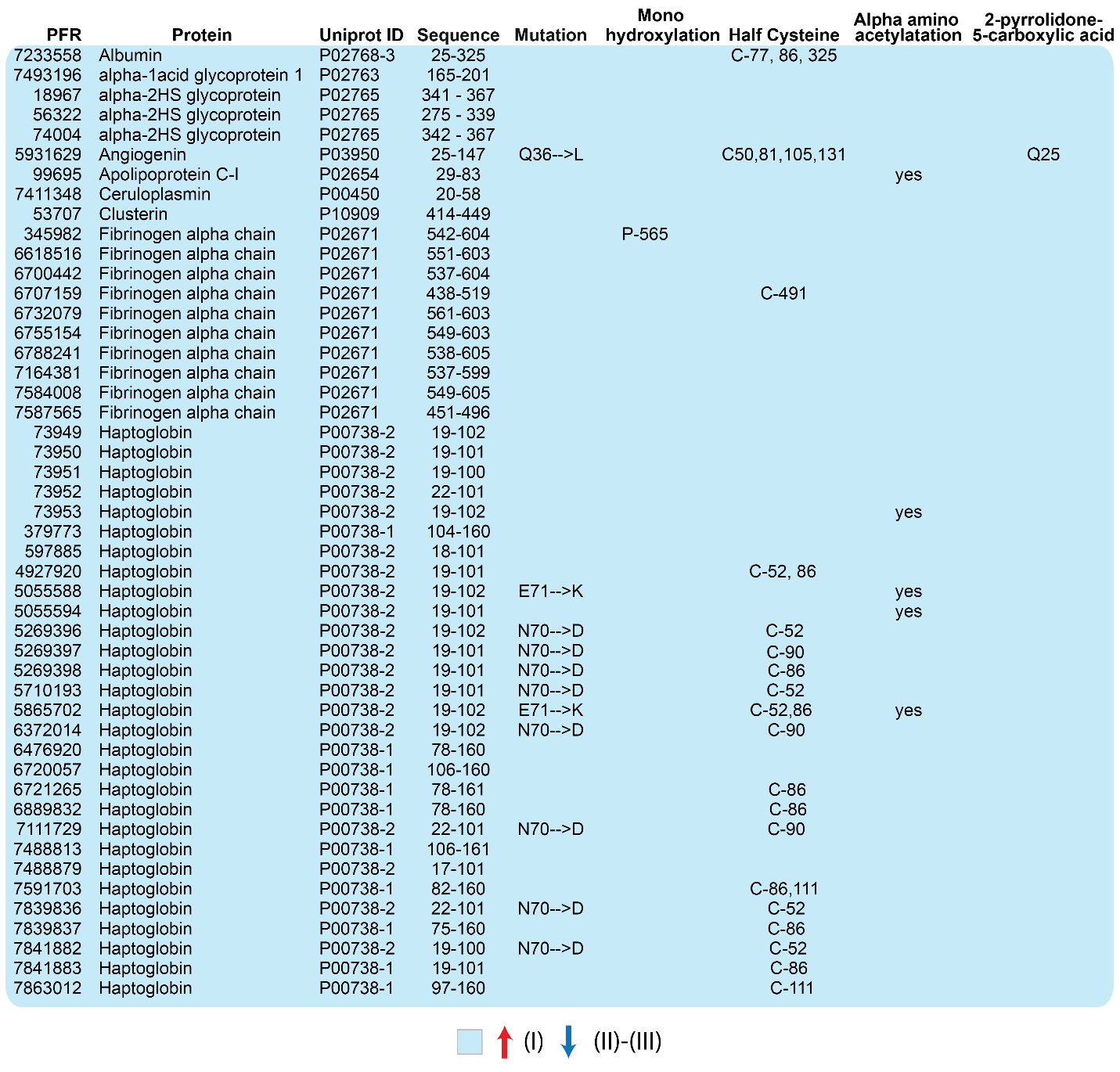


Fig. S12. Individual proteoforms upregulated in Stage I disease. Differentially expressed proteoforms (DEPs) significantly upregulated in stage (I) and downregulated in stages (II) and (III) (blue). Each proteoform is shown with its unique proteoform number, protein name,Uniprot ID, amino acid sequence relative to the Uniprot ID, any relevant mutations, and presence or absence of post-translational modifications. Colors are based on the proteoform signatures created in **Fig. 4**. Abbreviations:  PFR (proteoform).

*Note that half Cysteine could be artifact during the identification process.


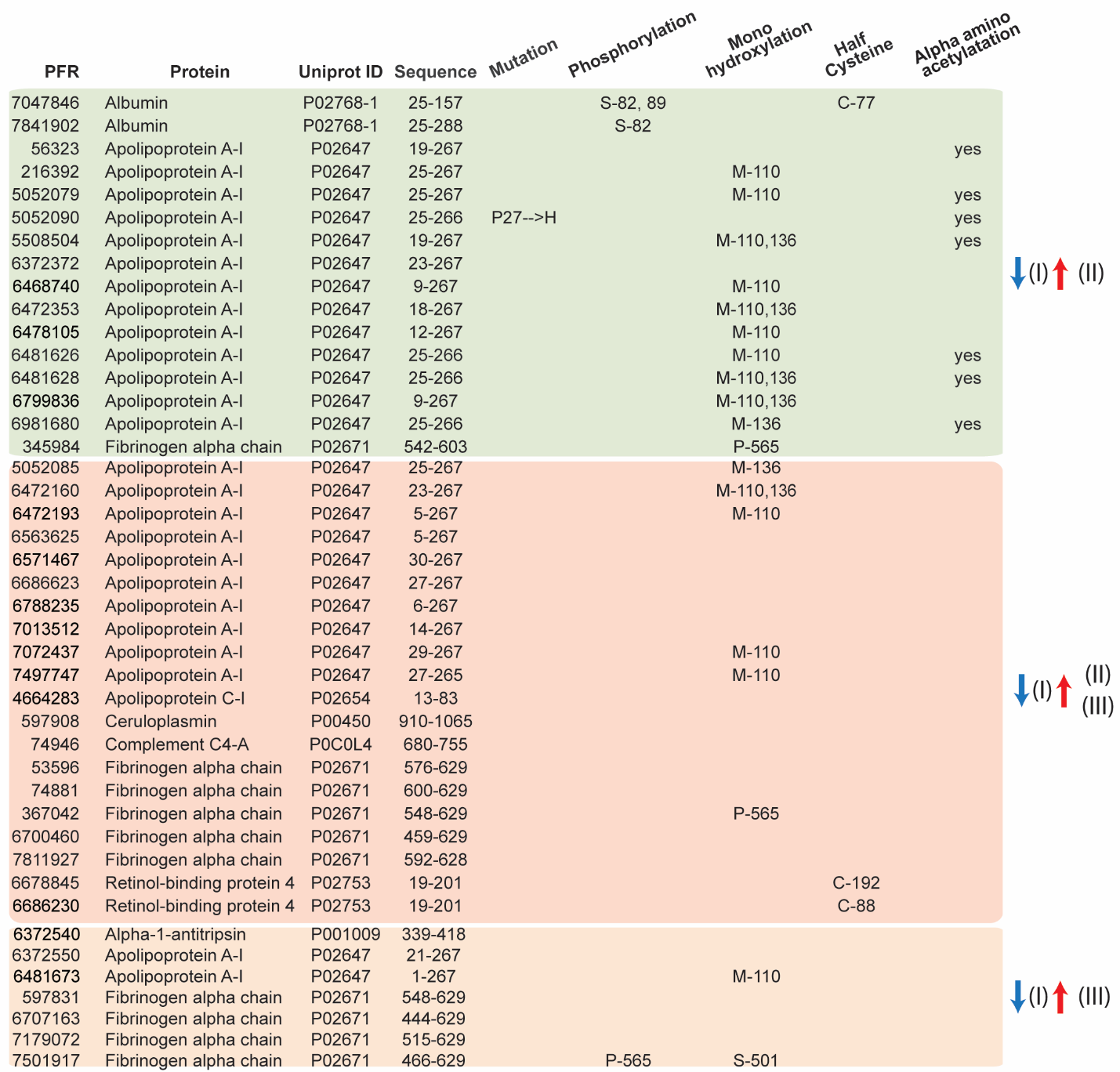


Fig. S13. Individual proteoforms upregulated in late-stage cirrhosis**.** Differentially expressed proteoforms (DEPs) significantly downregulated in stage (I) and upregulated in stage (II) (green), downregulated in stage (I) and upregulated in stages (II) and (III) (red), downregulated in stage (I) and upregulated in stage (III) (orange). Each proteoform is shown with its unique proteoform number, protein name and Uniprot ID, amino acid sequence relative to the Uniprot ID, and presence or absence of post-translational modifications. Colors are based on the proteoform signatures created in **Fig. 4**. Abbreviations:  PFR (proteoform).

*Note that half Cysteine could be artifact


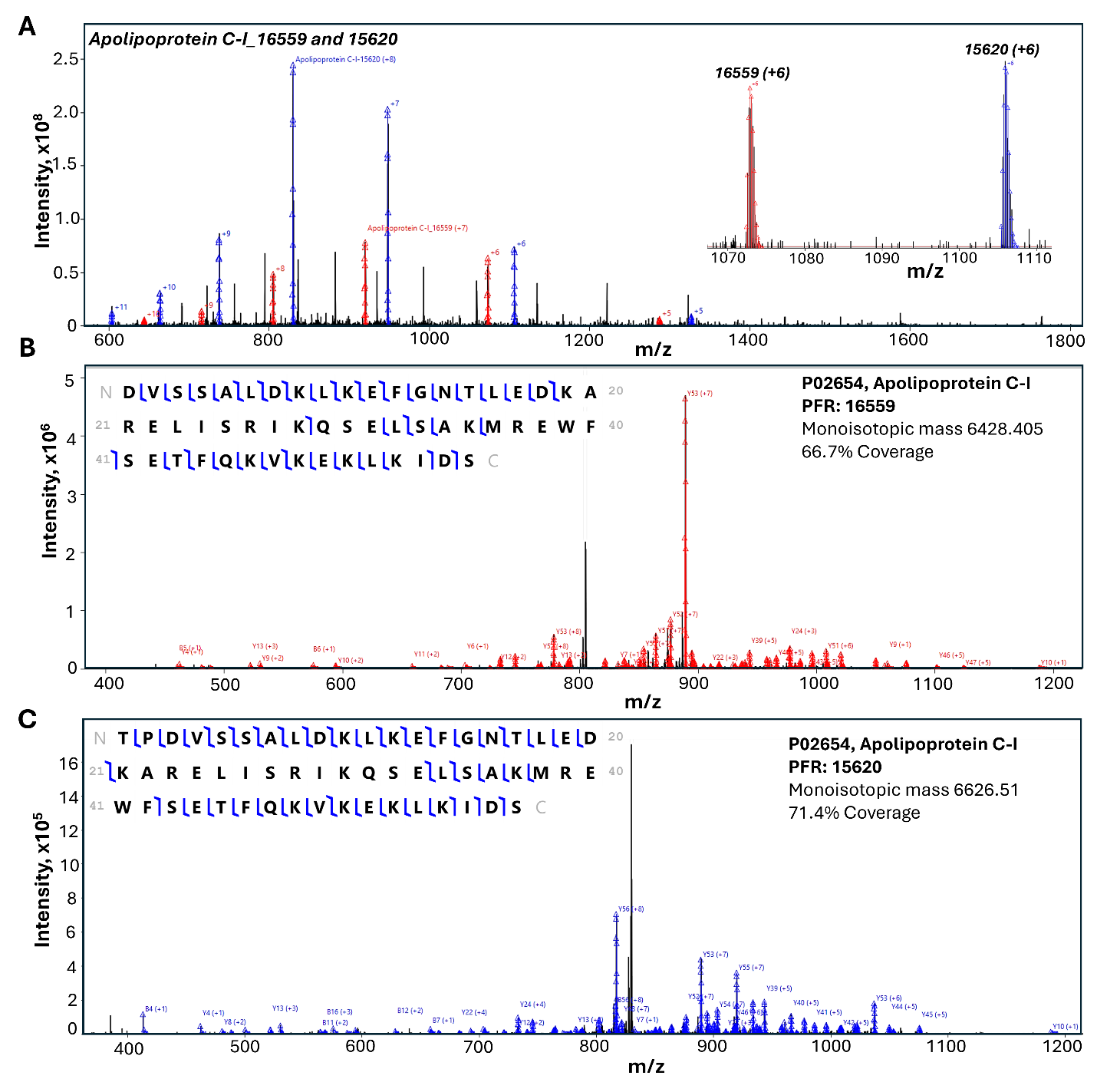


Fig. S14. MS1 and Tandem-MS/MS Fragmentation spectra of Apolipoprotein C-I**.** (A) MS1 spectra characteristic of the proteoforms PFR16559 (red), and PFR15620 (blue) (B) MS2 spectrum of PFR16559 showing sequence coverage of 66.7% mapping the N terminal truncation (C) MS2 spectrum of PFR15620 showing sequence coverage of 71.4% and mapping the canonical form of Apolipoprotein C-I.


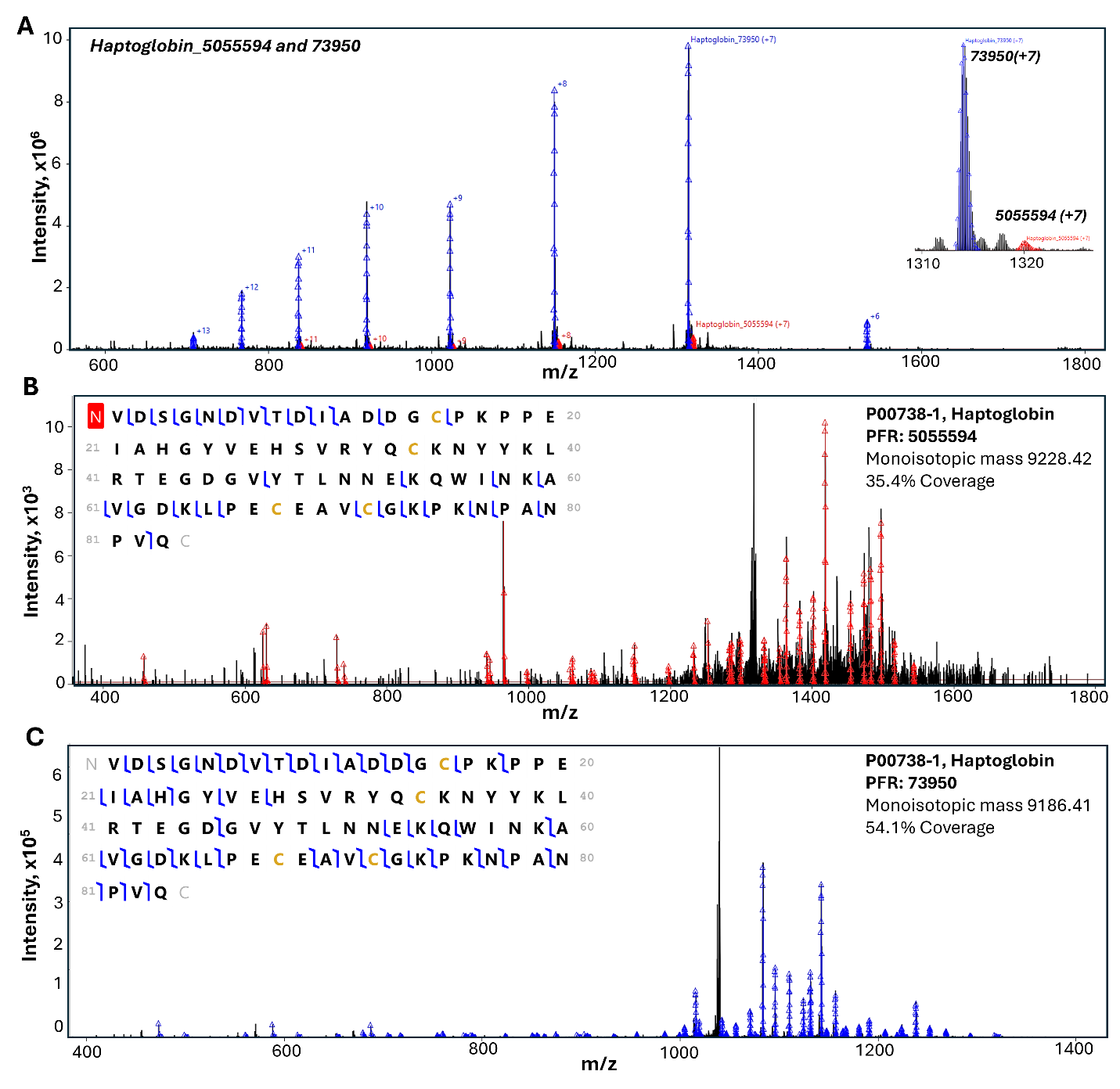


Fig. S15. MS1 and Tandem-MS/MS Fragmentation spectra of Haptoglobin**.** (A) MS1 spectra characteristic of the proteoforms PFR5055594 (red), and PFR73950 (blue) (B) MS2 spectrum of PFR5055594 showing sequence coverage of 35.4% mapping the acetylation at the N terminus. (C) MS2 spectrum of PFR73950 showing sequence coverage of 54.1% and mapping the N terminal truncation.


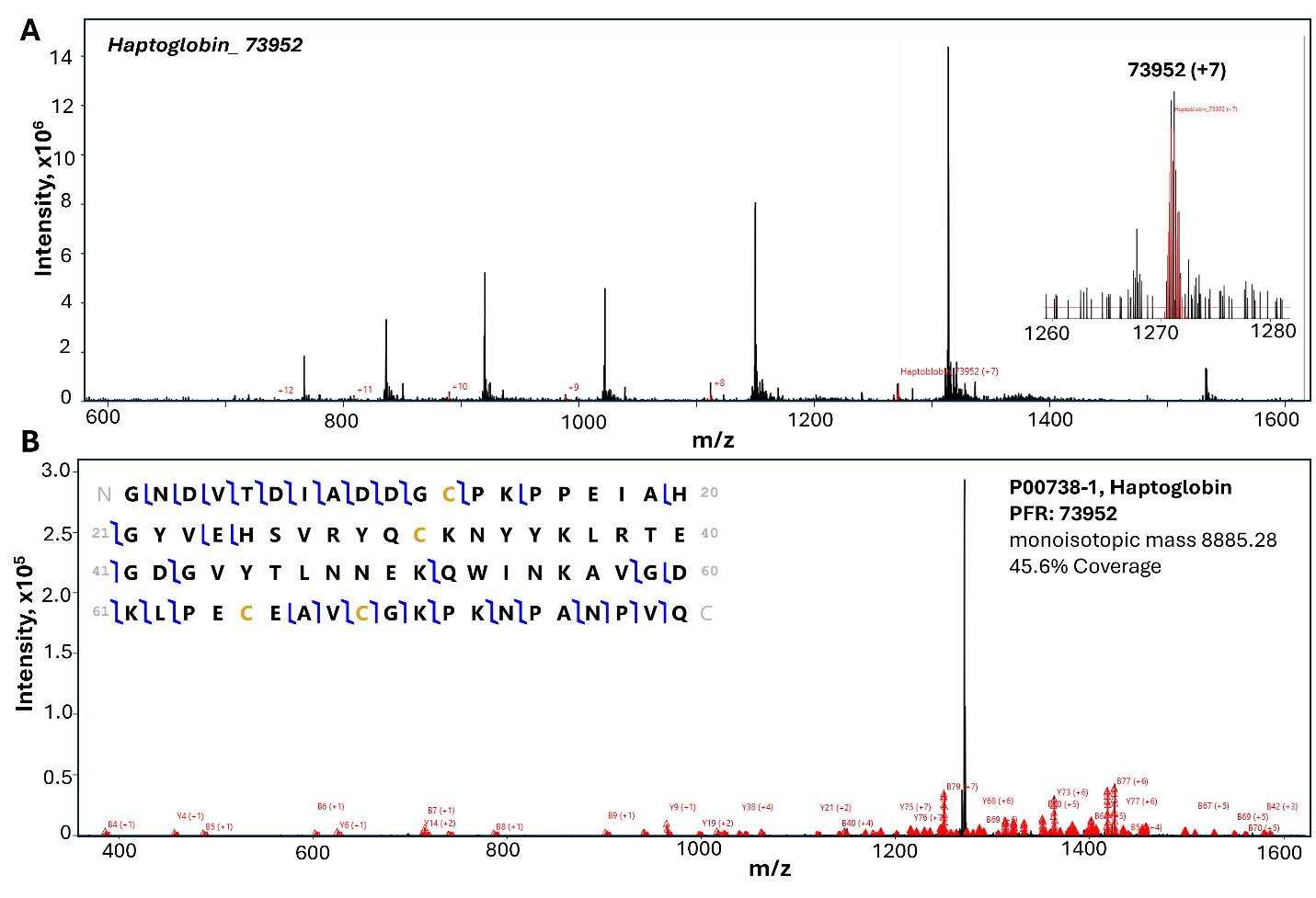


Fig. S16. MS1 and Tandem-MS/MS Fragmentation spectra of Haptoglobin**.** (A) MS1 spectra characteristic of the proteoforms PFR73952 (red) (B) MS2 spectrum of PFR73952 (red) showing sequence coverage of 45.6% mapping the N terminal truncation PFR.
